# Systemic infection exacerbates cerebrovascular dysfunction in Alzheimer’s disease

**DOI:** 10.1093/brain/awab094

**Published:** 2021-03-16

**Authors:** Daniel Asby, Delphine Boche, Stuart Allan, Seth Love, J Scott Miners

**Affiliations:** 1 Dementia Research Group, Bristol Medical School, University of Bristol, Bristol BS2 8DZ, UK; 2 Clinical Neurosciences, Clinical and Experimental Sciences, Faculty of Medicine, University of Southampton, Southampton S017 1BJ, UK; 3 Division of Neuroscience and Experimental Psychology, School of Biological Sciences, Faculty of Biology, Medicine and Health, The University of Manchester, Manchester Academic Health Science Centre, AV Hill Building, Manchester, M13 9PT, UK; 4 Geoffrey Jefferson Brain Research Centre, Manchester Academic Health Science Centre, Northern Care Alliance NHS Group and University of Manchester, Manchester, M13 9PT, UK

**Keywords:** Alzheimer’s disease, systemic infection, neuroinflammation, cerebral hypoperfusion, blood–brain barrier

## Abstract

We studied the effects of systemic infection on brain cytokine level and cerebral vascular function in Alzheimer’s disease and vascular dementia, in superior temporal cortex (Brodmann area 22) from Alzheimer’s disease patients (*n* = 75), vascular dementia patients (*n* = 22) and age-matched control subjects (*n* = 46), stratified according to the presence or absence of terminal systemic infection. Brain cytokine levels were measured using Mesoscale Discovery Multiplex Assays and markers of cerebrovascular function were assessed by ELISA. Multiple brain cytokines were elevated in Alzheimer’s disease and vascular dementia: IL-15 and IL-17A were maximally elevated in end-stage Alzheimer’s disease (Braak tangle stage V–VI) whereas IL-2, IL-5, IL12p40 and IL-16 were highest in intermediate Braak tangle stage III–IV disease. Several cytokines (IL-1β, IL-6, TNF-α, IL-8 and IL-15) were further raised in Alzheimer’s disease with systemic infection. Cerebral hypoperfusion—indicated by decreased MAG:PLP1 and increased vascular endothelial growth factor-A (VEGF)—and blood–brain barrier leakiness, indicated by raised levels of fibrinogen, were exacerbated in Alzheimer’s disease and vascular dementia patients, and also in non-dementia controls, with systemic infection. Amyloid-β_42_ level did not vary with infection or in association with brain cytokine levels. In controls, cortical perfusion declined with increasing IFN-γ, IL-2, IL-4, IL-6, IL-10, IL-12p70, IL-13 and tumour necrosis factor-α (TNF-α) but these relationships were lost with progression of Alzheimer’s disease, and with infection (even in Braak stage 0–II brains). Cortical platelet-derived growth factor receptor-β (PDGFRβ), a pericyte marker, was reduced, and endothelin-1 (EDN1) level was increased in Alzheimer’s disease; these were related to amyloid-β level and disease progression and only modestly affected by systemic infection. Our findings indicate that systemic infection alters brain cytokine levels and exacerbates cerebral hypoperfusion and blood–brain barrier leakiness associated with Alzheimer’s disease and vascular dementia, independently of the level of insoluble amyloid-β, and highlight systemic infection as an important contributor to dementia, requiring early identification and treatment in the elderly population.

## Teaser

Systemic infection exacerbates Alzheimer’s disease. In this postmortem study, Asby *et al*. provide evidence that it does so by raising the levels of multiple cytokines in the brain and by exacerbating cerebral hypoperfusion and vascular leakage in Alzheimer’s disease, independently of the level of insoluble amyloid-β42.

See Huuskonen *et al.* (doi:10.1093/brain/awab168) for a scientific commentary on this article.

## Introduction

Systemic infection may be associated with delirium and cognitive decline,^1^^,^^2^ and cognitive impairment is commonly observed in survivors of sepsis.^3^ Systemic infection is a risk factor for progression of Alzheimer’s disease^4^^,^^5^ and systemic infection and cognitive decline in Alzheimer’s disease are associated with raised serum IL-1β^6^ and TNF-α.^7^ Modelling of acute systemic infection in rodents induces microglial activation and elevated pro-inflammatory cytokine production (IL-1β, IL-6 and TNF-α), and exacerbates cognitive decline, neurodegeneration, and Alzheimer’s disease-like (amyloid-β and tau) pathology in mouse models.^8-11^ Post-mortem brain studies indicate that terminal systemic infection, recorded as the primary cause of death, is associated with activation of endothelial cells, perivascular macrophages and microglia,^12–14^ and we recently reported that the neuroinflammatory response to terminal systemic infection is modified in end-stage Alzheimer’s disease.^15^

Cerebrovascular dysfunction has been highlighted as a major contributor to cognitive decline and disease progression in Alzheimer’s disease (reviewed in Sweeney *et al*.^16^^,^^17^). Most patients with Alzheimer’s disease have post-mortem evidence of vascular disease,^18^ and clinical imaging and CSF biomarker studies have demonstrated blood–brain barrier breakdown^19^^,^^20^ and reduced cerebral blood flow up to 10–20 years before the onset of clinical symptoms.^21^ Disease modelling suggests that vascular dysfunction begins very early in the genesis of Alzheimer’s disease, around the time of initial amyloid-β accumulation.^22^ CSF changes in markers of pericyte injury and imaging of blood–brain barrier breakdown predicted cognitive decline in patients with mild cognitive impairment independently of changes in amyloid-β and tau.^20^^,^^23^

We previously demonstrated that biochemical changes associated with subacute and acute reduction in oxygenation of the cerebral cortex can be detected in post-mortem brain tissue in Alzheimer’s disease.^24^^,^^25^ These comprise a reduction in the level of myelin-associated glycoprotein (MAG) relative to proteolipid protein-1 (PLP1), two myelin proteins with similar long *in vivo* half-lives (several months) and post-mortem stability but with differential sensitivity to tissue hypoxia,^24–28^ and an increase in vascular endothelial growth factor-A (VEGF), induced by hypoxia-inducible factor-1α (HIF-1α).^29^ The extent of reduction in MAG:PLP1 ratio and elevation of VEGF correlate with: (i) amyloid-β_42_ level^28^; (ii) the level of fibrinogen (associated with blood–brain barrier leakiness); (iii) the decline in platelet-derived growth factor receptor-β (PDGFRβ) (reflecting loss of pericytes within the brain in Alzheimer’s disease); and (iv) the concentration of endothelin-1 (EDN1),^24^ a potent vasoconstrictor peptide that we previously showed to be elevated in Alzheimer’s disease.^30^^,^^31^

Systemic infection has a range of indirect effects on the extracranial vasculature. It increases the risk of coronary artery disease,^32^^,^^33^ renal stenosis, and peripheral atherosclerosis.^34^^,^^35^ Infection upregulates proatherogenic mediators including pro-inflammatory cytokines (IL-1β, IL-6), and cell adhesion molecules, such as intercellular adhesion molecule 1 (ICAM1) and vascular adhesion molecule 1 (VCAM1).^36^^,^^37^ A combination of elevated cytokine levels, increased blood viscosity, endothelial activation,^38^^,^^39^ smooth muscle cell proliferation, vascular remodelling and vasomotor dysfunction contribute to reduced perfusion, increased vascular permeability and increased risk of thrombosis in many tissues (reviewed in Pagnoux *et al*.^40^). Autoimmune mimicry can also contribute to remote vascular damage, e.g. in patients with periodontal disease.^41^

In view of the contribution of vascular dysfunction to the development and progression of Alzheimer’s disease, the accelerated cognitive decline in Alzheimer’s disease patients with systemic infection, and the known effects of infection and inflammation on extracranial vascular function, we hypothesized that the deleterious influence of systemic infection in dementia, particularly in Alzheimer’s disease, is at least partly mediated by exacerbated vascular dysfunction. We have used human post-mortem brain tissue to examine whether terminal systemic infection alters cytokine levels within the brain, and biochemical markers of cerebral oxygenation, blood–brain barrier function and other measures of vascular integrity and function, at different stages of Alzheimer’s disease as indicated by Braak tangle stage, in comparison with the effects in non-dementia controls and in cases with vascular dementia and mixed vascular and Alzheimer’s disease pathology. We show that systemic infection causes neuroinflammation and cerebral vascular dysfunction even in non-dementia controls, and exacerbates these processes in Alzheimer’s disease and vascular dementia.

## Materials and methods

### Study cohort

The use of human brain tissue for this study was approved by the management committee of the South West Dementia Brain Bank (Human Tissue Authority licence number 12273) under the terms of Bristol Research Ethics Committee approval (18/SW/0029). The right cerebral hemisphere had previously been fixed in buffered formalin for 3 weeks and was used for pathological assessment. The left cerebral hemisphere had been sliced and frozen at −80°C. Most brains were dissected within 72 h of death.

We studied 75 Alzheimer’s disease cases, 22 vascular dementia and 46 age-matched controls. A clinical history, which included post-mortem assessment and information on the death certificate, was used to subdivide cases according to whether systemic infection was or was not recorded as the primary cause of death in to the following groups: controls who died with (*n* = 22) or without systemic infection (*n* = 24); patients with Alzheimer’s disease who died with (*n* = 42) or without systemic infection (*n* = 33); and vascular dementia patients who died with (*n* = 7) or without systemic infection (*n* = 15).

Established internationally accepted neuropathological criteria were used to identify Alzheimer’s disease and vascular dementia cases. Alzheimer’s disease cases had a clinical diagnosis of Alzheimer’s disease during life and were subjected to detailed neuropathological assessment. We included cases with either intermediate or high Alzheimer’s disease neuropathological change that according to the NIA-AA guidelines^42^ was a sufficient explanation for the dementia. No other significant brain pathologies such as stroke, primary or metastatic brain tumour, or traumatic lesions were present in the Alzheimer’s disease cases. Cases with vascular dementia/mixed dementia had a clinical history of dementia, only occasional neuritic plaques, histopathological evidence of multiple infarcts/ischaemic lesions and moderate to severe atheroma and/or arteriosclerosis. In most cases there was no evidence of other disease likely to contribute to dementia, but in addition to the occasional neuritic plaques, three cases had moderate tangle pathology. Control brains were from individuals with no history of dementia, few or absent neuritic plaques, a Braak tangle stage of III or less, and no other neuropathological abnormalities. A summary of the demographic and clinical features of the cohorts are presented in Table 1. For this study, the superior temporal gyrus [Brodmann area (BA22)] was the brain area explored.

**Table 1:** Demographic and clinical features of the cohorts

**Cases**	Ctrl− (*n =* 24)	Ctrl+ (*n =* 22)	AD− (*n =* 33)	AD+ (*n =* 42)	VaD− (*n =* 15)	VaD+ (*n =* 7)
Gender	12F:12M	10F:12M	21F:12M	27F:15M	9F:6M	6F:1M
Age of death, years, mean ± SD	84.6 ± 7.1	85.8 ± 7.5	82.5 ± 6.9	81.9 ± 7.2	89.6 ± 7.3	83.7 ± 11.5
Age of AD onset, years, mean ± SD	n/a	n/a	74.0 ± 7.8	74.6 ± 9.0	78.6 ± 7.8	72 ± 15.9
Duration of AD, years, mean ± SD	n/a	n/a	7.6 ± 3.2	7.6 ± 4.2	9.3 ± 3.7	10 ± 6.7
Post-mortem delay, h, mean ± SD	37.9 ± 21.6	49.3 ± 21.9	40.4 ± 25.5	47.8 ± 21.9	40.3 ± 16.4	41.4 ± 23.3
Braak stage, *n*						
0–II	21	19	0	0	7	6
III–IV	3	3	8	8	5	1
V–VI	0	0	25	33	3	0
Cause of death, *n*						
Cardiovascular	19	3	11	1	3	0
Non-brain tumour	2	1	3	0	2	0
Bronchopneumonia	0	14	0	33	0	6
Urinary tract infection	0	2	0	3	0	1
Other, non-infection	3	0	19	0	10	0
Other, infection	0	2	0	5	0	0
*APOE* genotype						
*APOE* −/−	20	17	16	14	9	6
*APOE* ε4/−	3	5	11	20	5	1
*APOE* ε4/ε4	1	0	6	8	1	0

Braak stage was missing for a single AD+ case. *APOE* genotype (−) indicates absence of ε4 and possession of either *APOE* ε2 or 3. AD+/− = Alzheimer’s disease who died with/without systemic infection; Ctrl+/− neurologically or cognitively normal controls who died with/without systemic infection; VaD = vascular dementia who died with/without systemic infection; F = female; M = male; n/a = not applicable; SD = standard deviation.

### Multiplex analysis of brain cytokine and inflammatory markers in post-mortem brain tissue

Brain tissue (100 mg) was homogenized in 500 μl RIPA buffer (Thermo Fisher Scientific) supplemented with protease inhibitor cocktail (Complete mini; cat no. 04693124001; Roche) and phosphatase inhibitor cocktail (phosSTOP; cat no. 4906845001; Roche) using a Precellys^®^ automated tissue processor.

Inflammatory proteins were measured on the V-Plex MSD electrochemiluminescence multi-spot assay platform (MesoScale Diagnostics) using the V-Plex MSD Proinflammatory Human Protein Panel (cat. no. K15049D) and Cytokine Human Protein Panel (cat. no. K15050D), respectively. Brain homogenate (25 μl; 1:2 dilution) was used for each assay according to the manufacturer’s protocol, as previously described.^15^ Each plate was imaged on the Meso QuickplexSQ120 (MesoScale Discovery) according to manufacturer’s instructions for 384-well plates. Protein concentration was expressed in picograms per millilitre for each analyte after adjustment for total protein level, which was measured using the Total Protein kit (Sigma Aldrich).

### Biochemical assessment of vascular markers

Fresh frozen superior temporal cortex (BA22) (200 mg) was dissected and proteins were extracted in 1 ml of 1% sodium dodecyl sulphate lysis buffer, in a Precellys^®^ automated tissue processor (Stretton Scientific; Bertin Technologies) as previously described.^24^^,^^25^^,^^28^ Homogenates were centrifuged at 12 460*g* for 15 min at 4°C for and then aliquoted and stored at –80°C until required. Total protein was measured for all samples by use of Total Protein Kit according to manufacturer s guidelines (Sigma Aldrich).

### MAG:PLP1 ratio

The level of MAG was measured in homogenates diluted 1 in 10 in PBS, by in-house direct ELISA as previously described.^24–28^ A mouse monoclonal anti-MAG1 antibody (cat. no. ab89780; Abcam) diluted 1:1000 was used in the direct ELISA. PLP1 level was measured in brain tissue homogenates diluted 1 in 10 in PBS using a commercially available sandwich ELISA (cat. no. SEA417Hu, USCN), as described previously.^24–28^ The absorbance was measured at 450 nM in a FLUOstar^®^ Optima plate reader (BMG Labtech) after the addition of 2 N sulfuric acid. The concentration of MAG was interpolated from a serial dilution of recombinant human MAG (6.25–400 ng/ml) and adjusted for total protein level within each sample. The concentration of PLP1 was interpolated from a standard curve generated by serial dilution of recombinant human PLP1 (0.156–10 ng/ml) and adjusted for total protein. The ratio of MAG:PLP1 was calculated and is presented for each individual.

### VEGF ELISA

VEGF level was measured using the human VEGF-A ELISA kit (R&D Systems) as described previously.^24^^,^^25^^,^^28^ Brain tissue homogenates were diluted 1:10 in 1% BSA/PBS. Absorbance was measured at 450 nm in a FLUOstar^®^ Optima plate reader after the addition of 2 N sulfuric acid. VEGF concentration was interpolated from serial dilutions of recombinant human VEGF (2000–31.25 pg/ml) and adjusted for total protein level.

### Fibrinogen ELISA

Fibrinogen level was measured in brain tissue homogenates (2 μl + 248 μl PBS) using a commercially available sandwich ELISA (Human Fibrinogen ELISA kit, cat. No. EH3057, Wuhan Fine Biological Technology Co) as described previously.^28^ The concentration of fibrinogen was interpolated from measurements of serially diluted recombinant human fibrinogen (600–9.375 ng/ml) and adjusted for total protein level.

### PDGFRβ ELISA

PDGFRβ level was measured by sandwich ELISA (cat. No. DYC385, R&D Systems) as described previously.^28^ Absorbance was read at 450 nM following the addition of 2 N sulfuric acid, in a FLUOstar^®^ OPTIMA plate reader (BMG Labtech). The absolute concentration of PDGFRβ was interpolated from the standard curve for each case, derived from serial dilution of recombinant PDGFRβ, and adjusted for total protein.

### EDN1 ELISA

EDN1 was measured in tissue samples by a commercial sandwich ELISA (cat. no. QET00B, R&D Systems) as described previously.^24–26^ Each sample was individually diluted to achieve a final concentration of 1 mg/ml total protein and 50 μl of sample was added to each well. Relative luminescence was measured using a FLUOstar^®^ Optima plate reader (BMG Labtech). Absolute EDN1 level was interpolated from a standard curve generated by assaying serial dilutions of recombinant human EDN1 (0.343–250 pg/ml).

### Amyloid-β_42_ ELISA

Soluble and insoluble (guanidine‐extractable) fractions for amyloid-β_42_ measurement were prepared as reported previously.^28^ A commercial sandwich ELISA (R&D Systems) was used according to manufacturer’s instructions to measure amyloid-β_42_ in guanidine samples (diluted 1:2500 for Alzheimer’s disease samples and 1:625 for control and vascular dementia samples). Amyloid-β_42_ concentration was interpolated from serial dilutions of recombinant human amyloid-β_42_ (7.8–500 pg/ml) and corrected for sample dilution. Samples were measured in duplicate and the means calculated.

### Statistical analyses

The distribution of the data and identification of potential outliers were examined for all markers assessed by examination of quantile-quantile plots (not shown). To assess the effect of Alzheimer’s disease and/or systemic infection on inflammatory brain cytokines and vascular markers, we used both one-way and two-way ANOVAs, or their non-parametric equivalents (if the data were deemed to be not normally distributed), as appropriate. Data are presented as mean ± standard error of the mean (SEM). Pearson’s or Spearman’s tests were used as appropriate to assess linear correlation. All statistical analysis was performed with SPSS version 21 (SPSS, Chicago) and GraphPad Prism version 8 (GraphPad Software, La Jolla, CA). *P*‐values < 0.05 were considered statistically significant.

### Data availability

All data within the article are linked to the MRC UK-BBN by unique numeric MRC UK-BBN identifier (Supplementary Table 3). Further samples from the cases studied are available on request.

## Results

### Study cohort

We studied 143 cases comprising 75 Alzheimer’s disease (42 with terminal systemic infection and 33 without), 22 vascular dementia/mixed (seven with and 15 without terminal systemic infection) and 46 age-matched controls (22 with and 24 without terminal systemic infection). The age-at-onset of dementia, disease duration and Braak tangle stage for each of the six groups is shown in Table 1. Within each cohort, the distribution of Braak tangle stages was similar for the infection and non-infection groups. The groups were approximately matched for age-at-death and post-mortem delay. The gender split was approximately equal within the control cohort but skewed towards a higher proportion of females in the disease groups, as expected in the population. There was a higher proportion of *APOE* ε4 homozygotes and heterozygotes in the Alzheimer’s disease cohort but with a similar distribution of these alleles between the infection and non-infection Alzheimer’s disease groups.

Recorded causes of death in addition to dementia are listed in Table 1. Bronchopneumonia was the leading cause of death in the three groups with terminal systemic infection (33/42 Alzheimer’s disease cases, 6/7 vascular dementia cases and 14/22 controls); a smaller number of cases were recorded with terminal urinary tract infections (3/42 Alzheimer’s disease, 1/7 vascular dementia and 2/22 controls) or ‘other’ unclassified infections (2/22 Alzheimer’s disease, 5/42 Alzheimer’s disease, 0/7 vascular dementia). Causes of death in the non-infection cohort included systemic (non-stroke) cardiovascular disease (11/33 Alzheimer’s disease, 3/15 vascular dementia and 19/24 controls) and non-CNS tumours (3/33 Alzheimer’s disease, 2/15 vascular dementia, 2/24 controls).

### Brain cytokines are raised in Alzheimer’s disease and vascular dementia, and with systemic infection

**Figure 1:**
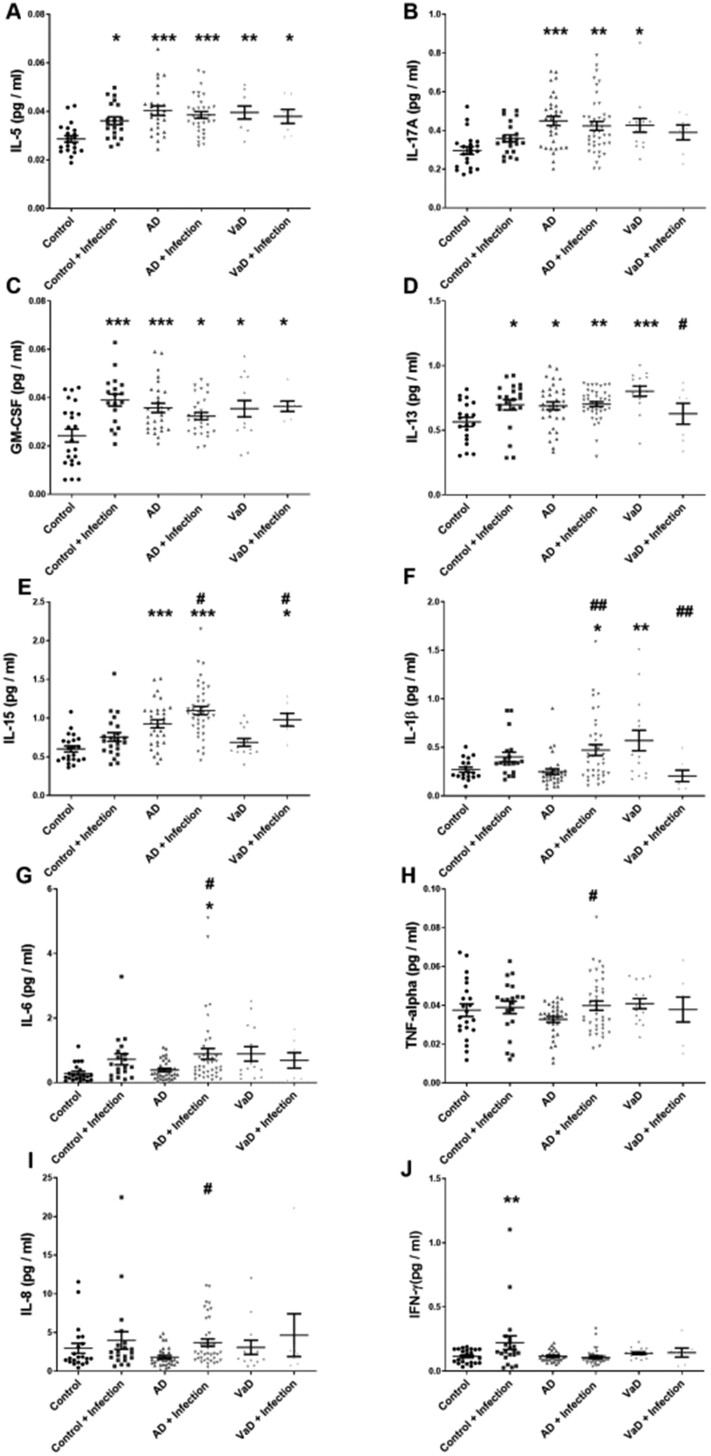
**Influence of systemic infection on brain cytokine levels in Alzheimer’s disease and vascular dementia.** Scatterplots showing cytokine levels in the superior temporal cortex (BA22) in post-mortem brain tissue in Alzheimer’s disease (AD) and vascular dementia (VaD) in the absence or presence of terminal systemic infection. Cytokine levels were measured using an MSD multiplex panel. Each point represents the mean of duplicate measurements for an individual. Horizontal bars indicate the cohort mean ± SEM. *Significant compared to age-matched controls; ^#^significant in association with terminal systemic infection in the same diagnosis group. *^/#^*P* < 0.05, **^/##^*P* < 0.01, ***^/###^*P* < 0.001

We performed one-way ANOVAs to assess differences in brain cytokine level between control, Alzheimer’s disease and vascular dementia groups after stratification according to the presence of infection (Fig. 1). In controls, IL-5, GM-CSF, IL-13 and IFN-γ were elevated in brains from control subjects with compared to those without infection (Fig. 1A, C, D and J). In Alzheimer’s disease cases, IL-15, IL-1β, IL-6, TNF-α, and IL-8 were higher in those with than without infection (Fig. 1E–I). In vascular dementia patients, IL-15 was higher in those with than without infection (Fig. 1E). In contrast, IL-13 and IL-1β levels were lower in vascular dementia patients with than without infection (Fig. 1D and F). We performed two-way ANOVAs to investigate differences in the interactions between dementia status and systemic infection (Supplementary Table 1). An interaction effect was observed between Alzheimer’s disease status and systemic infection indicating that GM-CSF, IL-17A, IFN-γ, and IL-12 were significantly altered by infection in Alzheimer’s disease (Supplementary Table 1). Interaction between vascular dementia status and systemic infection was seen for IL-13 and IL-1β (as shown by one-way ANOVA) and in addition, GM-CSF and IL-8. IL-2, IL-4, IL-7, IL-10, IL-12-23p40, IL-12p70, and IL-16 did not differ with dementia or in association with terminal systemic infection (Supplementary Fig. 1).

We assessed brain cytokine levels in relation to tangle progression, a proxy marker of disease stage in Alzheimer’s disease, in a combined Alzheimer’s disease and control cohort stratified into Braak stages 0–II (BS0–II), III–IV (BSIII–IV), and V–VI (BSV–VI). IL-15 and IL-17A were significantly elevated in end-stage disease (BSV–VI) compared to BS0–II (IL-15 was also elevated in BSIII–IV brains) (Supplementary Fig. 2AandB). IL-5 rose in mid-stage disease (BSIII–IV) only (Supplementary Fig.2C), and IL-2, IL-12p40 and IL-16 declined in end-stage disease (BSV–VI) (Supplementary Fig.2D–F). Several other brain cytokines—IL12-p70, IL-4, IL-7, IL-6 and IL-13—did not vary significantly with Braak stage, although the levels tended to be highest in BSIII–IV. GM-CSF and IL-1β did not vary with Braak stage and IFN-γ and IL-8 declined with increasing Braak stage (Supplementary Fig. 3).

### Cerebral hypoperfusion is exacerbated by systemic infection in controls and dementia

**Figure 2:**
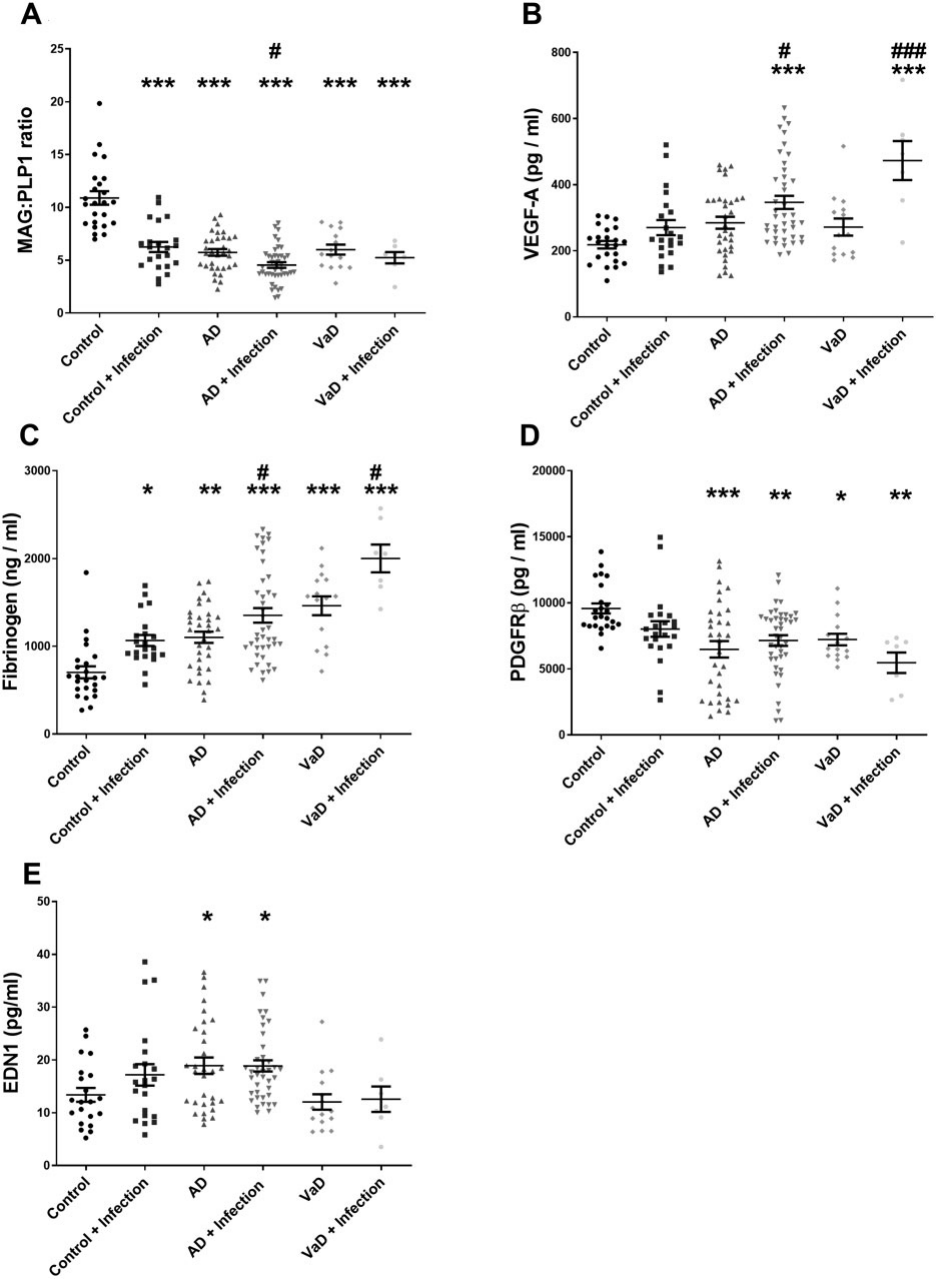
**Systemic infection and cerebrovascular dysfunction in Alzheimer’s disease and vascular dementia.** Scatterplots showing levels of several markers of cerebrovascular function/dysfunction in the superior temporal cortex (BA22) in post-mortem brain tissue in Alzheimer’s disease (AD) and vascular dementia (VaD) in the absence or presence of terminal systemic infection. Each point represents the mean of duplicate measurements for an individual. Horizontal bars indicate the cohort mean ± SEM. *Significant compared to age-matched controls. ^#^Significant in association with terminal systemic infection in the same diagnosis group. *^/#^*P* < 0.05, **^/##^*P* < 0.01, ***^/###^*P* < 0.001.

The MAG:PLP1 ratio was highly significantly reduced in Alzheimer’s disease and vascular dementia patients compared to age-matched controls (Supplementary Table 2). In a combined Alzheimer’s disease and control group, MAG:PLP1 was significantly reduced in BSIII–IV and BSV–VI compared to BS0–II (Supplementary Fig.4A). One-way ANOVA, to assess differences between control and disease groups after stratification according to the presence of infection, showed MAG:PLP1 to be reduced in controls with infection to a level comparable to that in Alzheimer’s disease or vascular dementia patients without infection (Fig. 2A). MAG:PLP1 was still further reduced in Alzheimer’s disease brains with than without infection but did not differ between vascular dementia patients with infection compared to those without (Fig. 2A). Two-way ANOVA revealed a highly significant interaction effect between infection and dementia status for MAG:PLP1 in both Alzheimer’s disease and vascular dementia patients (Supplementary Table 2).

VEGF, an independent marker of acute cerebral ischaemia,^24^^,^^25^ was highly significantly elevated in Alzheimer’s disease and vascular dementia compared to controls (Supplementary Table 2). Analysis of VEGF according to progression of tangle pathology in a combined cohort of Alzheimer’s disease and controls indicated that VEGF was higher in BSV–VI than BS0–II (Supplementary Fig. 4B). One-way ANOVA showed that VEGF was significantly elevated in Alzheimer’s disease with systemic infections versus those without, and vascular dementia pateints who died with systemic infections versus those without (Fig. 2B). Two-way ANOVA assessment of effects of interaction between infection and dementia status on VEGF level indicated that infection did not contribute significantly to the elevated VEGF in Alzheimer’s disease but did so in vascular dementia (interaction effect *P* = 0.008) (Supplementary Table 2).

### Blood–brain barrier leakiness is exacerbated by systemic infection in controls and dementia brains

Fibrinogen level within the brain, a marker of blood–brain barrier leakiness, was significantly higher in both Alzheimer’s disease and vascular dementia patients than control subjects (Supplementary Table 2), and significantly higher in vascular dementia than Alzheimer’s disease (Supplementary Table 2). Analysis of the effect of Braak tangle stage showed that fibrinogen was significantly higher in BSV–VI than in BS0–II (Supplementary Fig.4C). When cases were stratified according to systemic infection, a one-way ANOVA indicated that fibrinogen level was elevated in across control, Alzheimer’s disease and vascular dementia groups in the presence of systemic infection (Fig. 2C). A significant interaction effect of systemic infection on fibrinogen level was not, however, observed for controls versus Alzheimer’s disease and controls versus vascular dementia, in two-way ANOVAs, suggesting that the overall impact of systemic infection on blood–brain barrier leakiness was modest (Supplementary Table 2).

### PDGFRβ and EDN1 levels are altered in dementia and only modestly affected by systemic infection

The level of PDGFRβ, a protein expressed mainly by pericytes^28^ was significantly lower in Alzheimer’s disease and vascular dementia than controls (Fig. 2D andSupplementary Table 2). In relation to disease stage, PDGFRβ was lower in BSIII–IV (*P* < 0.05) and BSV–VI (*P* < 0.01) than in BS0–II (Supplementary Fig.4D). When Alzheimer’s disease patients and controls were stratified according to systemic infection, one-way ANOVA indicated that PDGFRβ did not differ between groups according to the presence of infection (Fig. 2D); however, a weak but significant effect of systemic infection on PDGFRβ was observed for Alzheimer’s disease patients versus controls (Supplementary Table 2; interaction effect, *P* = 0.039) but not for vascular dementia versus controls running a two-way ANOVA.

We have previously shown that cortical EDN1 level is elevated in Alzheimer’s disease.^24^ EDN1 level tended to be higher in Alzheimer’s disease, and lower in vascular dementia, compared to controls in the superior temporal cortex (Supplementary Table 2). When compared to controls without infection, EDN1 level was higher in Alzheimer’s disease groups irrespective of infection status (Fig. 2E).

### Amyloid-β_42_ in Alzheimer’s disease was unaltered by systemic infection

**Figure 3:**
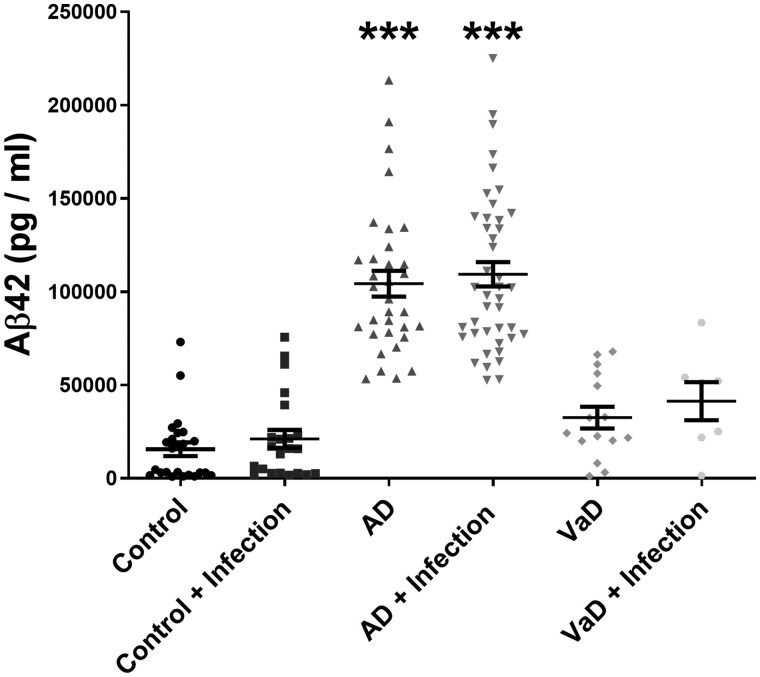
**Amyloid-β_42_ level is not influenced by terminal systemic infection.** Scatterplots showing amyloid-β_42_ (Aβ42) level in guanidine-HCl extracts (insoluble Aβ42) in superior temporal cortex (BA22) in Alzheimer’s disease (AD), vascular dementia (VaD) and age-matched controls, stratified for the absence or presence of terminal systemic infection. Each point represents the mean of duplicate measurements for an individual. Horizontal bars indicate the cohort mean ± SEM. *Significant compared to age-matched control. ***P < 0.001.

Amyloid-β_42_ level in guanidine-HCL extracts (i.e. in the insoluble pellet fraction) was significantly increased in Alzheimer’s disease, and to a much lesser extent in vascular dementia, compared to age-matched controls (Fig. 3). Amyloid-β_42_ did not vary according to the presence of systemic infection in any of the groups and did not correlate with brain cytokine levels (data not shown).

### Cerebral perfusion is related to brain cytokine levels in early stages of Alzheimer’s disease

**Figure 4:**
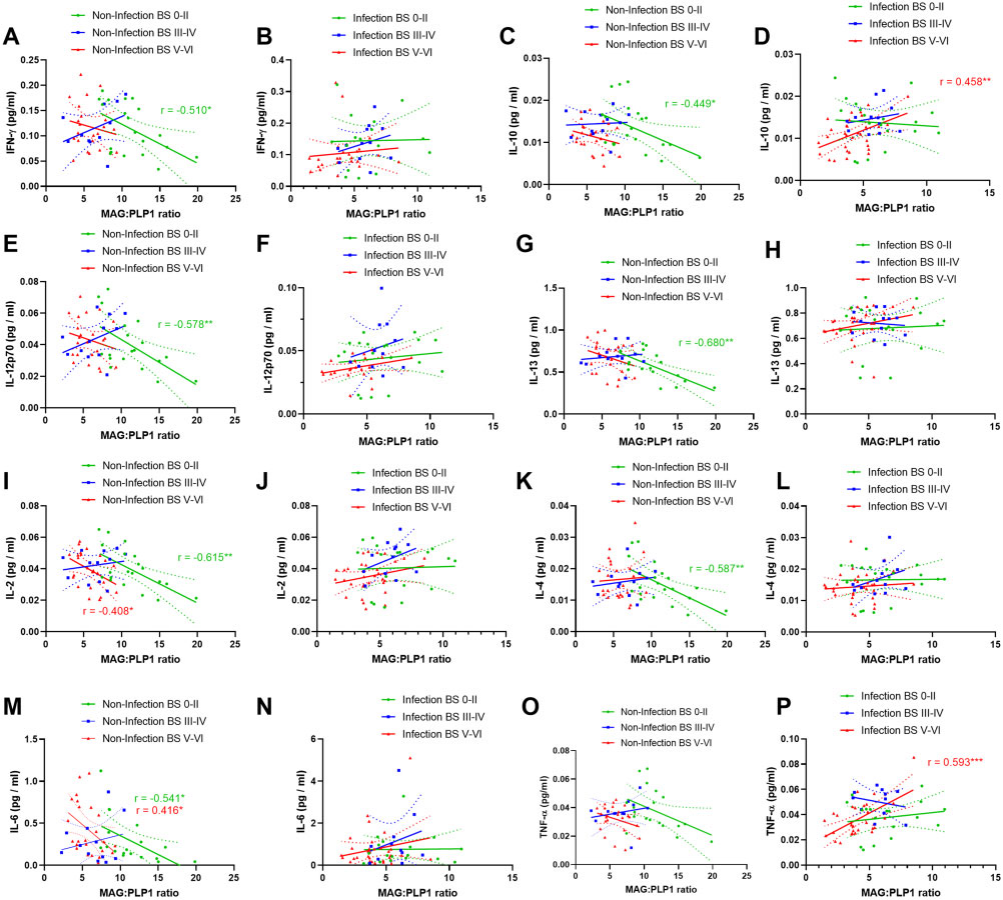
**Relationship between biochemical markers of brain perfusion and brain cytokines: influence of Braak stage and infection.** In brains with minimal tangle pathology (BS0–II) but not more advance disease, MAG:PLP1 correlated negatively with a large number of brain cytokines. With infection, these correlations were lost, even in BS0–II disease. The best-fit linear regression lines and 95% confidence intervals are shown.

In the absence of infection or substantial Alzheimer’s disease tangle pathology (i.e. in BS 0–II), cortical perfusion, as indicated by MAG:PLP1, correlated negatively with the levels of several cytokines (IFN-γ, IL-2, IL-12p70, IL-6, IL-10, IL-13, IL-4) but with few exceptions this correlation was lost with infection or progression of tangle pathology (Fig. 4). Notably, TNF-α and IL-10 correlated positively with MAG:PLP1 in BSV–VI only (Fig. 4).

**Figure 5:**
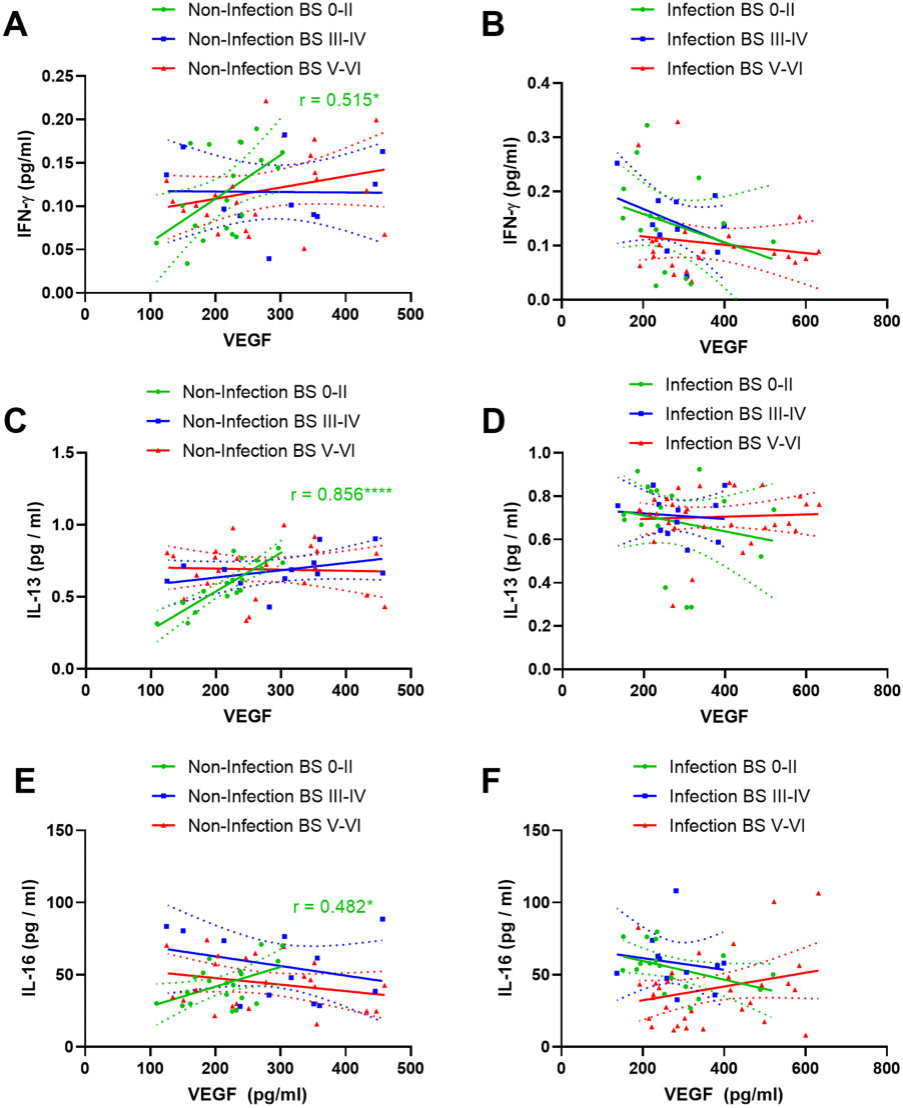
**Relationship between VEGF and brain cytokines: influence of Braak stage and infection.** In brains with minimal tangle pathology (BS-0–II) but not more advanced disease, VEGF correlated positively with several brain cytokines. This relationship was lost even in BS0–II in the presence of systemic infection. The best-fit linear regression lines and 95% confidence intervals are shown.

Similarly, VEGF correlated positively with IFN-γ, IL-13 and IL-16 in BS0–II in the absence of systemic infection or substantial tangle pathology but the association was again lost in BSIII–IV and V–VI, and even sooner, in BS0–II, in cases with infection (Fig. 5).

MAG:PLP1 and VEGF showed the expected negative correlation, as previously reported^24^^,^^26^ in BS0–II, but this relationship was lost in BSIII–IV and BSV–VI and in all brains with systemic infection (Supplementary Fig. 5).

### Blood–brain barrier leakiness is related to elevated IL-1β in early and amyloid-β_42_ in late disease stage

As previously reported in the precuneus, fibrinogen level in the superior temporal cortex was inversely corelated with markers of cerebral hypoperfusion (reduced MAG:PLP1 and elevated VEGF) (SupplementaryFig. 6A and B) and positively correlated with amyloid-β_42_ in controls and Alzheimer’s disease cases, but not vascular dementia (SupplementaryFig.6C). Fibrinogen also correlated with reduced pericyte marker, PDGFRβ, level in controls (SupplementaryFig.6D).

**Figure 6:**
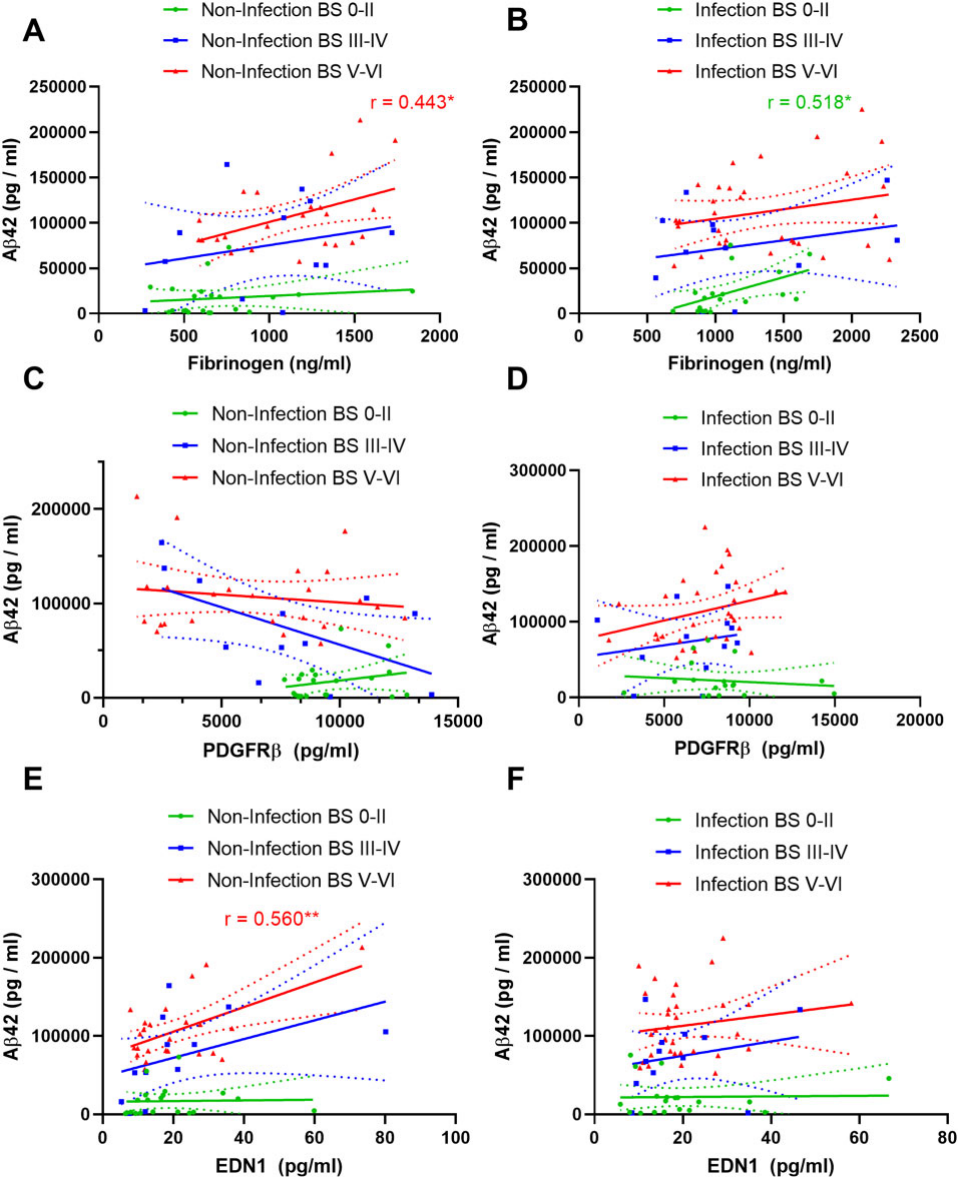
**Relationships of PDGFRβ, fibrinogen and endothelin-1 with amyloid-β_42_: influence of Braak stage and infection.** (**A** and **B**) Fibrinogen correlated positively with amyloid-β42 in BSV–VI without and BS0–II with systemic infection. (**C** and **D**) PDGFRβ correlated negatively with amyloid-β_42_ (Aβ42) approaching significance (*r* = −0.0568; *P* = 0.0541) in BSIII–IV in the absence, and positively in BSV–VI in the presence, of terminal systemic infection in superior temporal cortex. (**E** and **F**) EDN1 correlated positively with MAG:PLP1 in BSV–VI in BSV–VI only without infection. The best-fit linear regression lines and 95% confidence intervals are shown.

Brain fibrinogen correlated positively with IL-1β in the early stages of disease (BS0–II and BSIII–IV) – the relationship was lost in BSV–VI cases and with systemic infection as early as BS0–II (Supplementary Fig. 7AandB). Fibrinogen also correlated weakly with IL-13 in BSIII–IV without infection but not when infection was present (Supplementary Fig. 7CandD). Fibrinogen correlated positively with amyloid-β_42_ in Braak tangle stage V–VI only; the relationship between fibrinogen and amyloid-β_42_ at each stage of disease was not substantially affected by systemic infection (Fig. 6A and B).

Brain fibrinogen level was also raised in Alzheimer’s disease in individuals homozygous for *APOE* ε4, as was EDN1 level (SupplementaryFig.8). MAG:PLP1 tended to be lower, and VEGF and PDGFRβ tended to be higher, in individuals heterozygous or homozygous for *APOE* ε4 but these differences did not reach statistical significance. Except for IL-6 and IL-13, brain cytokine level was not related to possession of *APOE* ε4 in either controls or Alzheimer’s disease brains (SupplementaryFig.9).

### PDGFRβ and EDN1 level are only modestly affected by systemic infection

PDGFRβ tended to decline with increasing amyloid-β_42_ in Alzheimer’s disease brains in the absence of infection and to increase slightly in the presence infection but none of these trends was significant (Fig. 6C and D). PDGFRβ correlated negatively in BSIII–IV and positively in BSV–VI with several cytokines (IL-10, IL-12, IL-13, IL-2, IL-4 and TNF-α) but only in the absence of infection (Supplementary Fig. 10).

EDN1 correlated with amyloid-β_42_ in BSV–VI, only in those cases without infection (*r* = 0.560, *P* < 0.01) (Fig. 6E). Systemic infection had only a modest effect on this relationship (Fig. 6f).

In the absence of systemic infection, EDN1 correlated positively with the level of IL-15, IL-5, IL-1β and IL-17A in BSIII–IV or BSV–VI disease. These relationships were lost in systemic infection (Supplementary Fig. 11). TNF-α was an exception, in that the level did not correlate with EDN1 in the absence of infection; however, in cases with terminal infection, TNF-α showed a weak negative correlation with EDN1 in BS0–II disease and a strong positive correlation in advanced Alzheimer’s disease (BSV–VI).

## Discussion

In this post-mortem study, we show that brain cytokine levels and markers of cerebrovascular dysfunction in the superior temporal gyrus are exacerbated in the presence of terminal systemic infection in Alzheimer’s disease and vascular dementia, and in healthy age-matched controls. The influence of systemic infection on brain cytokines and vascular function varied with the stage of disease (as indicated by Braak tangle stage); brain cytokines were often highest at BSIII–IV and markers of cerebral vascular function were often impaired at this early to intermediate stage of disease. Our data indicate that systemic infection, independently of amyloid-β_42_ level, contributes to raised brain cytokine level and vascular insufficiency, particularly cerebral hypoperfusion and blood–brain barrier leakiness in early Alzheimer’s disease. Markers of cerebral hypoperfusion and blood–brain barrier breakdown were associated with elevated levels of brain cytokines in early disease (BS0–II) but these relationships were often lost in the presence of systemic infection or disease pathology. In contrast, systemic infection only contributed modestly to disease-related changes in late-stage disease and the expression of the vasoconstrictor, EDN1, and the pericyte marker, PDGFRβ, were associated with amyloid-β_42_ at a later stage of disease. These data indicate that the contribution of systemic infection to brain cytokine expression and vascular insufficiency varies according to disease stage: cerebral hypoperfusion and blood–brain barrier is exacerbated by infection and is related to elevated brain cytokine expression at an early stage of Alzheimer’s disease, independently of amyloid-β, whereas pericyte loss, raised EDN1, and further blood–brain barrier breakdown, are related to amyloid-β accumulation in late-stage disease.

Systemic infection has long been recognized as a cause of cognitive impairment and delirium. Alzheimer’s disease patients with raised serum levels of pro-inflammatory cytokines IL-1β and TNF-α are indeed at increased risk of subsequent cognitive decline.^6^^,^^7^ The level of IL-1β within the brain is elevated by peripheral administration of endotoxins, simulating sepsis, suggesting that systemic infection may exacerbate already present brain inflammatory responses in Alzheimer’s disease.^8–11^ In our previous post-mortem study, we found evidence of downregulation of pro-inflammatory cytokines in brain tissue when infection occurred in end-stage Alzheimer’s disease.^15^ Here, we assessed the impact of systemic infection on brain cytokine expression in superior temporal cortex from brains representing the full spectrum of Alzheimer’s disease progression, from BS0–II to BSV–VI, as well as from patients with neuropathologically confirmed vascular dementia. Levels of some cytokines (IL-15 and IL-17A) were highest in brains from Alzheimer’s disease patients with BSV–VI disease. IL-15 is a pleiotropic cytokine that is highly expressed in activated astrocytes and contributes to disease pathology in brain ischaemia^43^ and multiple sclerosis.^44^ IL-15 level is raised in the CSF in relation to cognitive impairment and disease progression in Alzheimer’s disease.^45^ IL-17, released from activated microglia, is associated with neurodegeneration *in vitro*,^46^ and with disease pathology and cognitive decline in a mouse model of amyloid-β accumulation.^47^ It may also have a role in the recruitment of peripheral neutrophils in Alzheimer’s disease.^48^ IL-17 has been found to drive tau hyperphosphorylation,^49^ and it was notable that IL-17 level was highest in BSV–VI brains.

For many cytokines, (IL-5, IL-2, IL-12p40 and IL-16), however, the level was highest in BSIII–IV disease, suggesting perhaps that the deleterious effects of systemic infection on the brain are likely to be maximal at an early to intermediate stage of Alzheimer’s disease. This is consistent with clinical observations,^45^ brain imaging of microglia^50^^,^^51^ and post-mortem observation of activated microglia in controls with amyloid-β pathology, potentially reflecting early-mid stage disease^52^ indicating that neuroinflammation occurs at an early presymptomatic stage in Alzheimer’s disease and contributes to cognitive decline and disease progression. Brain cytokine levels were unrelated to insoluble amyloid-β_42_ level, and amyloid-β_42_ levels were unchanged by infection, possibly suggesting that the impact of systemic infection of brain cytokines occurred independently of amyloid-β pathology.

Cerebrovascular dysfunction, associated with reduced cerebral blood flow^21^ and cerebrovascular damage, including blood–brain barrier leakiness, is apparent not only in vascular dementia but also from an early stage in the development of Alzheimer’s disease.^16^^,^^17^ Recent high-resolution imaging studies have revealed leakiness of the blood–brain barrier in the hippocampus in pre-symptomatic Alzheimer’s disease.^19^ Later studies by the same group indicated that blood–brain barrier breakdown precedes changes in the levels of amyloid-β and tau in the CSF in the earliest stages of Alzheimer’s disease.^20^ These vascular abnormalities are accelerated with possession of *APOE* ε4,^23^ in keeping with earlier post-mortem studies indicating that pericyte loss and blood–brain barrier breakdown are more pronounced in individuals with *APOE* ε4.^28^^,^^53^ Elevated levels of endothelin-1 (EDN1) in Alzheimer’s disease may contribute to cerebral hypoperfusion via contraction of smooth muscle cells on penetrating arteries and arterioles^24^ and pericyte dysfunction, an essential component of the neurovascular unit, contributes to blood flow dysregulation and as mentioned, blood–brain barrier breakdown. Pericyte injury upon exposure to amyloid-β peptides or hypoxia in vitro,^54^ resulting in shedding and elevated CSF level of soluble PDGFRβ (sPDGFRβ) in Alzheimer’s disease^55^ is related to blood–brain barrier damage.^19^^,^^20^

Our recent post-mortem studies indicate that biochemical markers of pathological hypoperfusion and reduced oxygenation of the cerebral cortex in Alzheimer’s disease and vascular dementia are associated with elevated levels of amyloid-β_42_, EDN1, and fibrinogen, and reduced PDGFRβ.^24^^,^^25^^,^^28^ The level of fibrinogen, a marker of blood–brain barrier leakiness, correlated with that of amyloid-β_42_ and was inversely related to the concentration of PDGFRβ and to the MAG:PLP1 ratio.^28^ Raised CSF markers of cerebrovascular function, including YKL-40, ICAM1, VCAM1 and VEGF receptor 1 (Flt1), are elevated in presymptomatic Alzheimer’s disease in association with cognitive decline and markers of cortical thinning^45^ and correlated with CSF tau, as was also the case for CSF levels of soluble PDGFRβ (a marker of pericyte injury).^55^ Here, we show that MAG:PLP1 and PDGFRβ were significantly reduced, at an early stage i.e. BSIII–IV, in Alzheimer’s disease indicating vascular dysfunction from an early to intermediate stage of disease. We found that terminal systemic infection exacerbated cortical perfusion and blood–brain barrier function not only in Alzheimer’s disease but also in healthy controls. Cerebral hypoperfusion and blood–brain barrier, associated with systemic infection, was likely independent of amyloid-β_42_ (which was unaltered in late-stage Alzheimer’s disease in the presence of infection). Indeed, in cases with no or minimal Alzheimer’s disease pathology (BS0–II), MAG:PLP1 declined and VEGF and fibrinogen increased by magnitudes similar to those in end-stage Alzheimer’s disease in control donors with terminal infection. In contrast, systemic infection appeared to have a more modest effect on EDN1 and PDGFRβ level. We previously showed that EDN1, fibrinogen and PDGFRβ are related to amyloid-β_42_ level.^24^ Here we show that PDGFRβ and EDN1 (and to some extent fibrinogen) levels are related to amyloid-β_42_ in Braak tangle stage III–IV and V–VI disease but not BS0–II, perhaps reflecting a threshold effect of classical Alzheimer’s disease pathological processes on the regulation of these vasoactive molecules. Together, these data indicate a complex relationship between cerebrovascular dysfunction in Alzheimer’s disease, likely to involve multiple mediators, which is both dependent and independent of amyloid-β and tau depending on stage of disease.

It is likely that cerebrovascular dysfunction associated with systemic infection is related to the systemic effects of circulating cytokines as well as localized brain-expressed pro-inflammatory cytokines. In this study, we found that in the absence of significant brain pathology or systemic infection, the expression of several brain cytokines was higher in brains that were less well perfused, i.e. with lower MAG:PLP1. The relationships were lost in the presence of systemic infection and disease pathology, suggesting that these pathological processes overwhelm the normal, relatively subtle, inflammatory responses to reductions in perfusion. Cytokines play a multifactorial role in vascular injury, mediating both vasoconstriction and dilatation (reviewed in Vila and Salaices^56^). Systemic inflammation is associated with cerebral hypoperfusion, via EDN1-mediated vasoconstriction.^57^ Reduced regional blood flow in the brain in rats exposed to LPS, to model septicaemia that resulted in microglial activation and neuronal loss, was associated with enhanced transcription of several cytokines and chemokines including TNF-α, IL-1β, TGF-β and MCP-1 within the brain.^58^

Experimental chronic cerebral hypoperfusion caused an increase in pro-inflammatory cytokines,^59^ and IL-1β infusion exacerbated cerebral hypoperfusion.^60^ Several cytokines modulate signalling pathways that regulate vascular tone, some increasing the production of vasodilators (NO, PGI_2_), and other pro-inflammatory cytokines, such as TNF-α, upregulating the expression of the potent vasoconstrictors, Ang-II and EDN1,^61^ which are also elevated in Alzheimer’s disease.^24^^,^^25^^,^^30^ Our findings indicate that this complex interrelationship between ischaemia, cytokine production and cerebral perfusion is altered by systemic infection and influenced by the severity of Alzheimer’s disease pathology, with evidence that aberrant patterns of response of several cytokines in hypoperfused tissue varied according to Braak stage. The patterns of response of some cytokines, such as IFN-γ and IL-12p70, were most abnormal for infection in BS0–II disease, whereas for others, such as IL-10 and TNF-α, the relationships between cytokines and markers of perfusion were most abnormal for infection in BSV–VI disease.

Systemic infection and neuroinflammation alter blood–brain barrier permeability (reviewed in Varatharaj and Galea^62^). Blood–brain barrier leakiness is often associated with raised levels of pro-inflammatory cytokines, such as IL-1β, TNF-α, IL-6 and IL-2^63–67^ and pro-inflammatory cytokines directly influence blood–brain barrier permeability in rodent endothelial cell cultures^68^ and isolated cerebral microvessels from sheep.^69^ For instance, elevated endothelial expression of IL-6, in response to TNF-α, reduces the expression of tight junction proteins including cadherin, occludin and claudin-5 in human brain endothelial cells^70^^,^^71^ and TNF-α induces pericytes to produce MMP9, which increases blood–brain barrier leakiness.^72^ Brain fibrinogen was related to IL-1β (and IL-13 to a smaller extent) in early and intermediate stages of disease (BS0–II and III–IV) but the relationship was lost in end stage disease, or with infection. A recent study revealed that IL-1β released by activated microglia disrupts astrocytic regulation of blood–brain barrier permeability, by suppressing astrocytic expression of sonic hedgehog protein.^73^ Other cytokines, including IL-17A, were shown to reduce or redistribute tight junction proteins in a human cerebral microvascular cell line (hCMEC/D3).^74^ Opening of the blood–brain barrier allows peripheral cytokines to enter the brain,^58^ further compromising cerebral vascular function. In late, stage disease, BSV–VI, fibrinogen level was associated with insoluble amyloid-β_42_ level and was only modestly affected by systemic infection. The effects of systemic infection^75^ on intravascular fibrinogen, and stalling of blood flow in brain capillaries may also have contributed to the elevated brain fibrinogen level. However, the rise in intracerebral fibrinogen was of the order of 100% in our cases with terminal infection, whereas even chronic systemic inflammation (e.g. in rheumatoid arthritis) causes a rise in intravascular fibrinogen of about 50%^76^. Second, correction for variations in haemoglobin concentration (a proxy indicator of blood content) made only a small (up to a few per cent) difference to the raw measurements of fibrinogen (not shown) in our study, so it is unlikely that changes in intravascular fibrinogen level made more than a modest contribution to the increase in intracerebral fibrinogen in the cases with systemic infection.

In conclusion, we have found that systemic infection is associated with elevated levels of multiple cytokines within the brain and exacerbates hypoperfusion and blood–brain barrier leakage at an early/intermediate disease stage possibly independently of amyloid-β_42_. PDGFRβ, a marker of pericytes, EDN1 levels, and fibrinogen level, were associated with amyloid-β_42_ level at a more advanced stage of disease and appeared to be only modestly affected by systemic infection. The retrospective, observational, post-mortem nature of this study imposes limitations on the interpretation of our findings, particularly insofar as the evidence is circumstantial and does not inform directly on causality or underlying mechanisms. The extent to which our findings are relevant to the progression of disease in a chronic condition with an extended prodromal phase remains to be determined. However, we know that cerebrovascular dysfunction is a strong predictor of cognitive decline and demonstrable in the early stages of dementia, perhaps independent of amyloid-β and tau, and our observations are in keeping with studies in animal models of amyloid-β accumulation which indicate that both systemic infection and cerebral hypoperfusion exacerbate disease progression and pathology. Preservation of proteins is always a concern in post-mortem studies, but to assess vascular function we have used biochemical markers that we have previously shown that to be stable for up to 72 h under simulated post-mortem conditions.^27^^,^^31^

In conclusion, our data are in keeping with a range of previous experimental and observational studies of the relationship between systemic inflammation and cytokine levels within the brain; the effects of cytokines on microvascular perfusion and permeability; the association of both hypoperfusion and blood–brain barrier breakdown with cognitive impairment; and the deleterious impact of systemic infection on the progression of dementia in Alzheimer’s disease. In Alzheimer’s disease, vascular dysfunction is strongly associated with the level of insoluble amyloid-β_42_. Our findings suggest that systemic infection exacerbates Alzheimer’s disease mostly through additive, cytokine-mediated vascular dysfunction that is independent of the level of insoluble amyloid-β_42_ in the early stages of disease.

## Supplementary Material

awab094_Supplementary_Data

## Abbreviations

BS: Braak stage
