# Supplementary material for: Systemic infection exacerbates cerebrovascular dysfunction in Alzheimer’s disease
Source: Brain. 2021 Mar 16;144(6):1869–83. doi: 10.1093/brain/awab094 (PMC8320299; doi:10.1093/brain/awab094)
Supplement: awab094_Supplementary_Data [file awab094_supplementary_data.pdf]

Supplementary Tables and Figures

**Con Vs AD**	**Diagnosis Con Vs AD**	**Systemic Infection**	**Interaction effect**		**Con Vs VaD**	**Diagnosis Con Vs VaD**	**Systemic Infection**	**Interaction effect**
**IL-5**	p < 0.01	NS	NS		**IL-5**	p < 0.05	NS	NS
**IL-17A**	p < 0.0001	NS	p < 0.05		**IL-17A**	p < 0.01	NS	NS (0.070)
**GM-CSF**	NS	0.022	p < 0.0001		**GM-CSF**	NS	0.022	0.032
**IL-13**	p < 0.05	p < 0.05	NS		**IL-13**	NS (0.098)	NS	p < 0.01
**IL-15**	p <0.00001	p < 0.01	NS		**IL-15**	p < 0.05	p < 0.01	NS
**IL-1B**	NS	p < 0.01	NS		**IL-1B**	NS	NS	p < 0.001
**IL-6**	NS	p < 0.01	NS		**IL-6**	NS	NS	NS (0.066)
**TNF-a**	NS	NS	NS		**TNF-a**	NS	NS	NS
**IL-8**	NS (0.077)	p < 0.05	NS		**IL-8**	NS (0.064)	NS	0.025
**IFN-g**	0.014	p < 0.05	p < 0.01		**IFN-g**	NS	NS	NS
**IL-2**	NS	NS	NS		**IL-2**	NS	NS	NS
**IL-4**	NS	NS	0.043		**IL-4**	NS	NS	NS
**IL-7**	NS	NS	NS		**IL-7**	NS	NS	NS
**IL-10**	NS (0.051)	NS	NS		**IL-10**	NS	NS	NS
**IL-12/p40**	p < 0.05	NS	NS (0.055)		**IL-12/p40**	NS	NS	NS
**IL-12/p70**	NS	NS	p < 0.05		**IL-12/p70**	NS	NS	NS
**IL-16**	NS	NS	NS		**IL-16**	NS	NS	NS

Supplementary Table 1. The contribution of systemic infection on brain cytokine levels in Alzheimer's disease (AD) and Vascular dementia (VaD). 2-WAY ANOVAs were performed to identify disease-specific differences and identify whether systemic infection significantly altered brain cytokine levels in Con Vs AD and Con Vs VaD, indicated by the interaction effect. p-values < 0.05 were considered statistically significant (p-values approaching p < 0.05 are shown in parenthesis).

**Con Vs AD**	**Diagnosis Con Vs AD**	**Systemic infection**	**Interaction effect**		**Con Vs VaD**	**Diagnosis Con Vs VaD**	**Systemic Infection**	**Interaction effect**
**MAG:PLP1**	p < 0.00001	p < 0.00001	p <0.00001		**MAG:PLP1**	p < 0.0001	p < 0.001	p < 0.01
**VEGF-A**	P < 0.001	P < 0.01	NS		**VEGF-A**	p < 0.0001	p < 0.0001	p < 0.01
**Fibrinogen**	p < 0.001	p < 0.001	NS		**Fibrinogen**	p < 0.00001	p < 0.00001	NS
**PDGFRβ**	p < 0.001	NS	p < 0.05		**PDGFRβ**	p < 0.001	p < 0.01	NS
**EDN1**	NS	NS	NS		**EDN1**	P < 0.05	NS	NS

Supplementary Table 2. The contribution of systemic infection on vascular dysfunction in the temporal cortex (BA22) in Alzheimer's disease (AD) and Vascular dementia (VaD). 2-WAY ANOVAs were performed to identify differences in vascular marker between disease groups and whether systemic infection contributes to disease-related changes in cerebrovascular markers in Control (Con) Vs AD and Control (Con) Vs VaD. p values < 0.05 were considered statistically significant (p-values approaching p < 0.05 are shown in parenthesis).

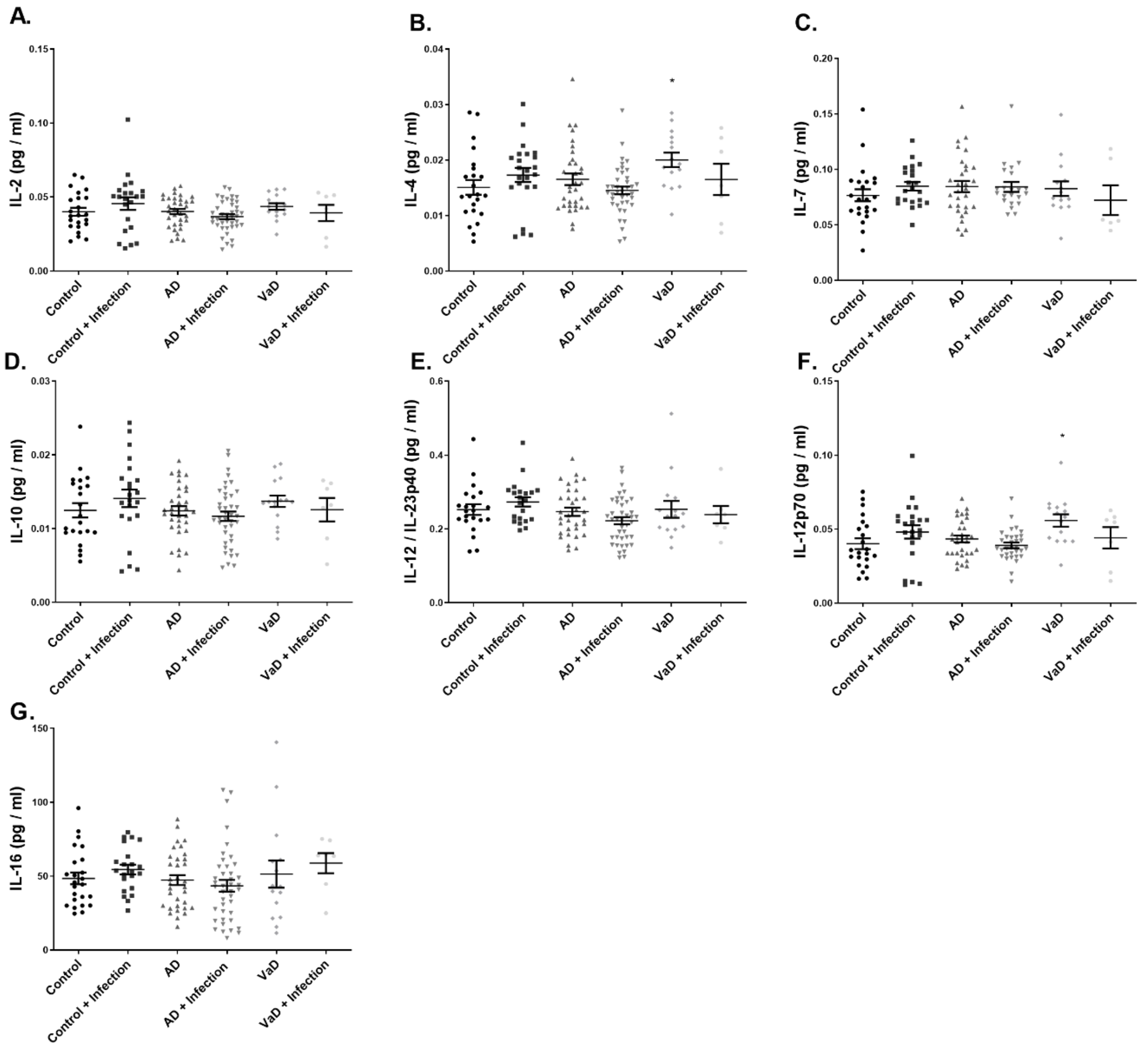

Supplementary Figure 1. Influence of systemic infection on brain cytokine levels in Alzheimer's disease and vascular dementia. Scatterplots showing cytokine levels in the superior temporal cortex in post-mortem brain tissue in Alzheimer's disease (AD) and vascular dementia (VaD) in the absence or presence of terminal systemic infection. Cytokine levels were measured using an MSD multiplex panel. Each point represents the mean of duplicate measurements for an individual. Horizontal bars indicate the cohort mean ± SEM.

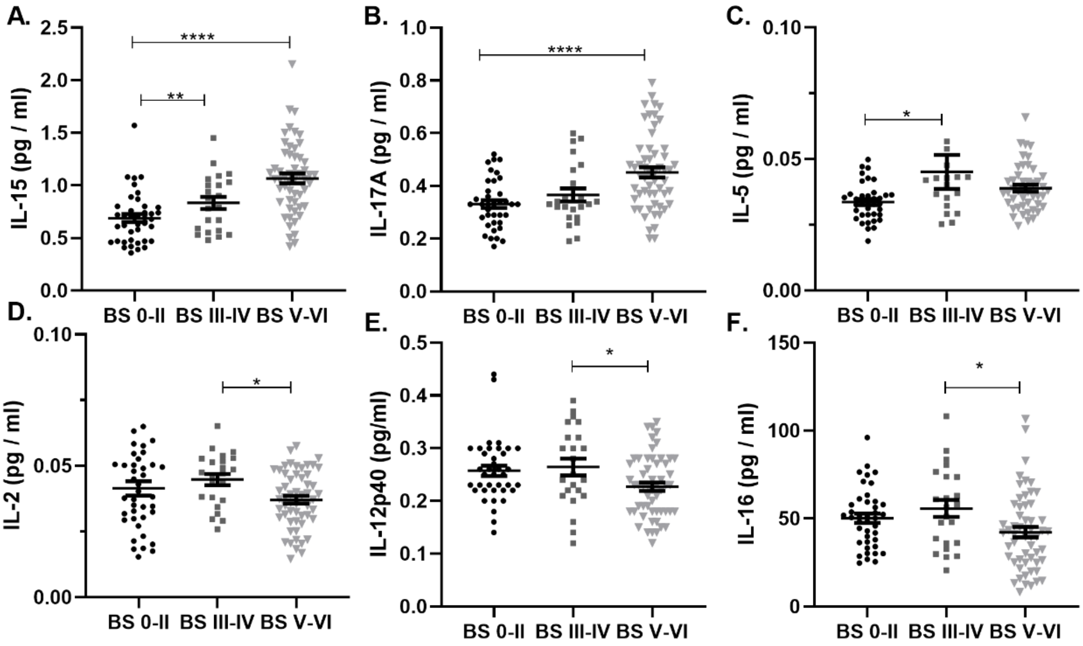

Supplementary Figure 2. Brain cytokine levels in relation to Braak tangle stage.

Scatterplots showing cytokine levels in the superior temporal cortex (BA22) in post-mortem brain tissue in a combined AD and control cohort stratified according to Braak tangle stage (BS): BS 0-II, BS III-IV and BS V-VI. Cytokine levels were measured using an MSD multiplex panel. Each point represents the mean of duplicate measurements for an individual. Horizontal bars indicate the cohort mean ± SEM. * $p < 0.05$, ** $p < 0.01$, *** $p < 0.001$, **** $p < 0.0001$

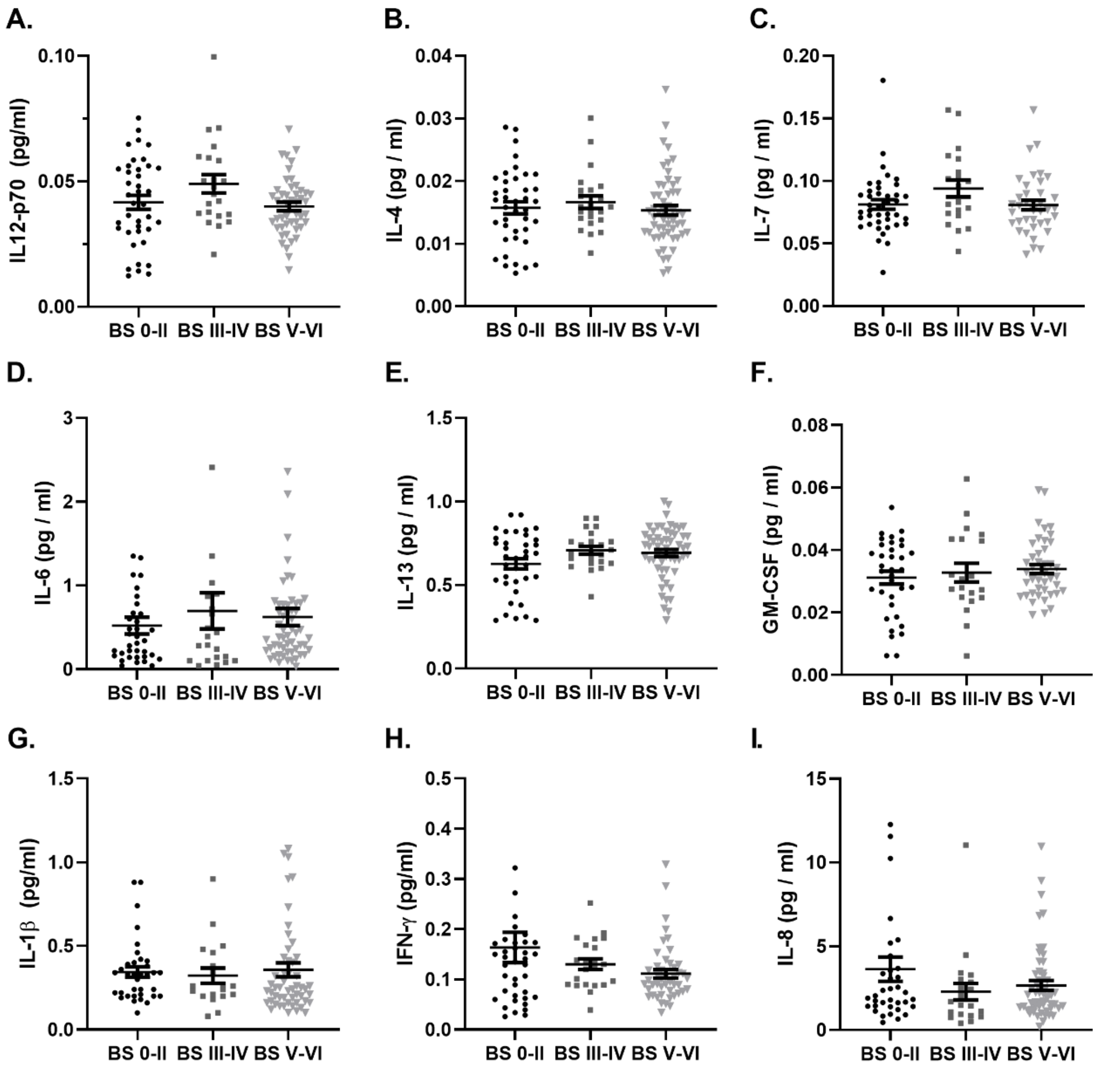

Supplementary Figure 3. Influence of Braak tangle stage pathology on brain cytokine levels. Scatterplots showing cytokine levels in the superior temporal cortex in post-mortem brain tissue in a combined Alzheimer's disease and age-matched control cohort. Each point represents the mean of a duplicate measurement for an individual. Horizontal bars indicate the cohort mean ± SEM

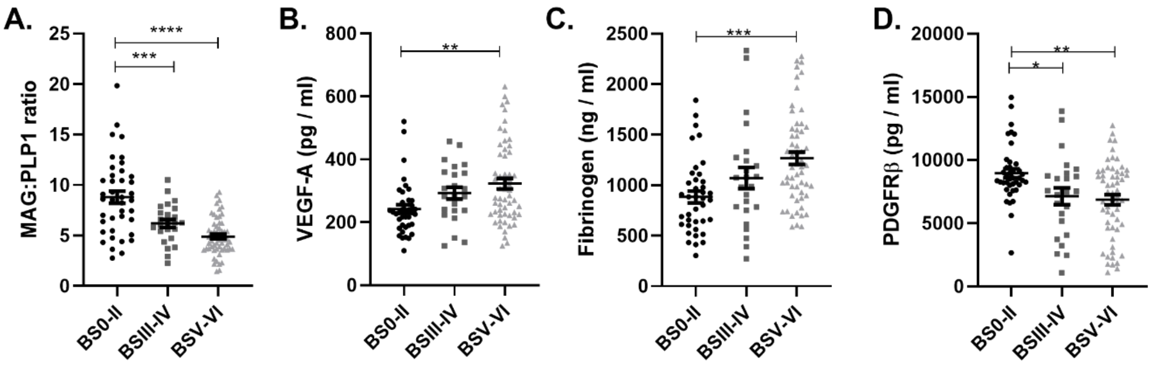

Supplementary Figure 4. Vascular markers in relation to Braak tangle. Scatterplots showing (A) MAG:PLP1 ration (B) VEGF (C) Fibrinogen and (D) PDGFRβ in AD and control cases divided into Braak tangle stage (BS). Each point represents the mean of a duplicate measurement for an individual. Horizontal bars indicate the cohort mean ± SEM. * $p < 0.05$ ** $p < 0.01$ *** $p < 0.001$ **** $p < 0.0001$

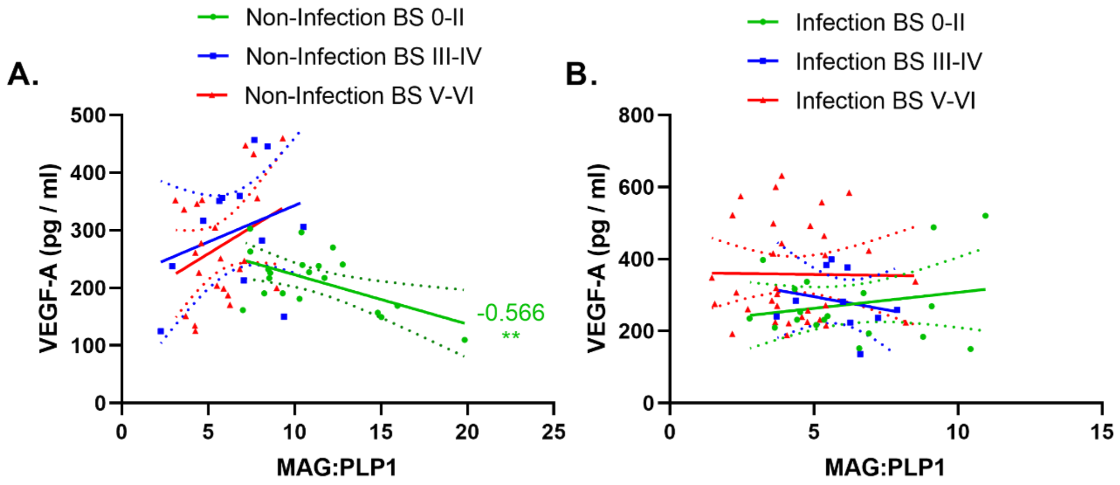

Supplementary Figure 5. Relationship between MAG:PLP and VEGF: influence of infection. In brains with minimal tangle pathology (BS 0-II) but not more advanced disease, VEGF correlated negatively with MAG:PLP1 in superior temporal cortex. With infection, this correlation was lost even in BS 0-II disease. The best-fit linear regression lines and 95% confidence intervals are shown.

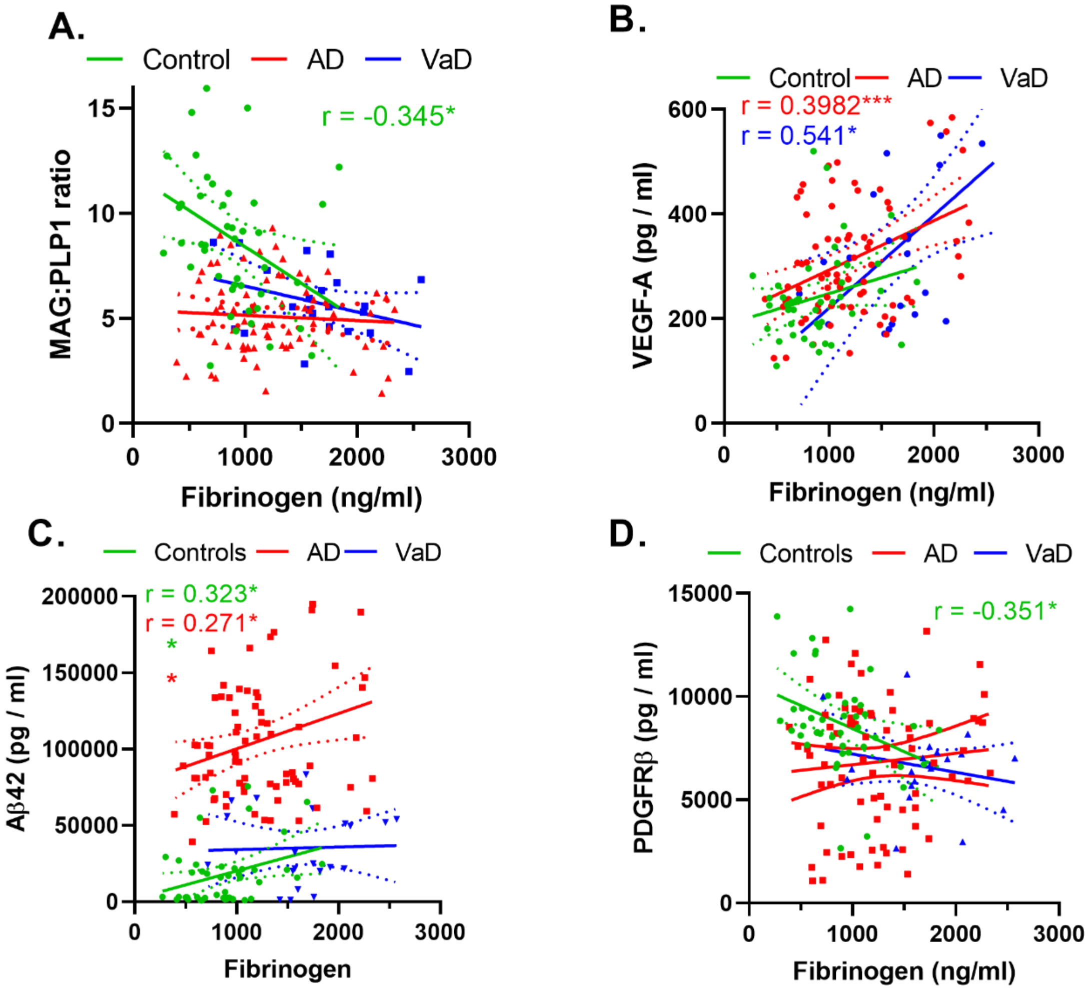

Supplementary Figure 6: Relationships between brain fibrinogen level and markers of brain perfusion (MAG:PLP1), pericyte content (PDGFRβ) and Aβ42 in dementia. (A-B) Brain fibrinogen correlated with MAG:PLP1, a marker of reduced oxygenation and positively with VEGF, a marker of acute ischaemia, in AD and VaD. (C) Fibrinogen was increased in relation to Aβ42 in AD only. (D) Fibrinogen was related to lower PDGFRβ level in controls. The best-fit linear regression lines and 95% confidence intervals are shown.

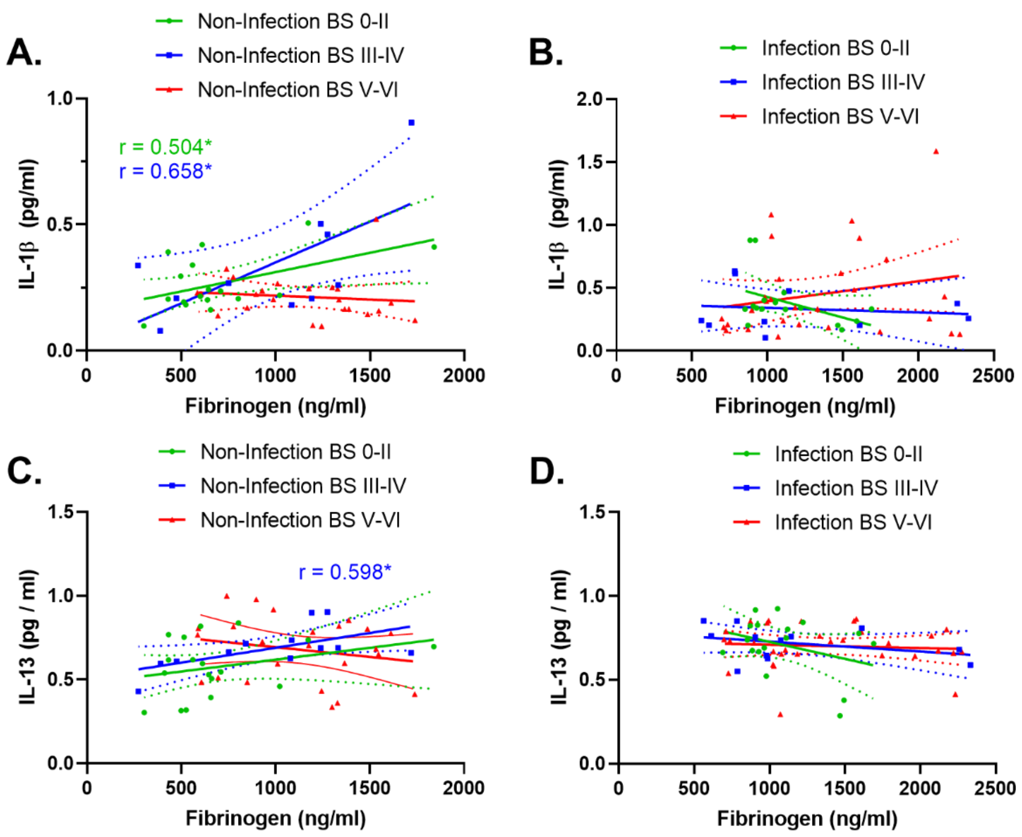

Supplementary Figure 7. Relationship between fibrinogen and brain cytokines: influence of Braak stage and infection. Most brain cytokines did not correlate with fibrinogen level, however, fibrinogen correlated with IL-1β in BS III-IV and V-VI in cases without systemic infection but not with infection. Fibrinogen correlated positively with IL-13 in BS III-IV without but not with infection. The best-fit linear regression lines and 95% confidence intervals are shown.

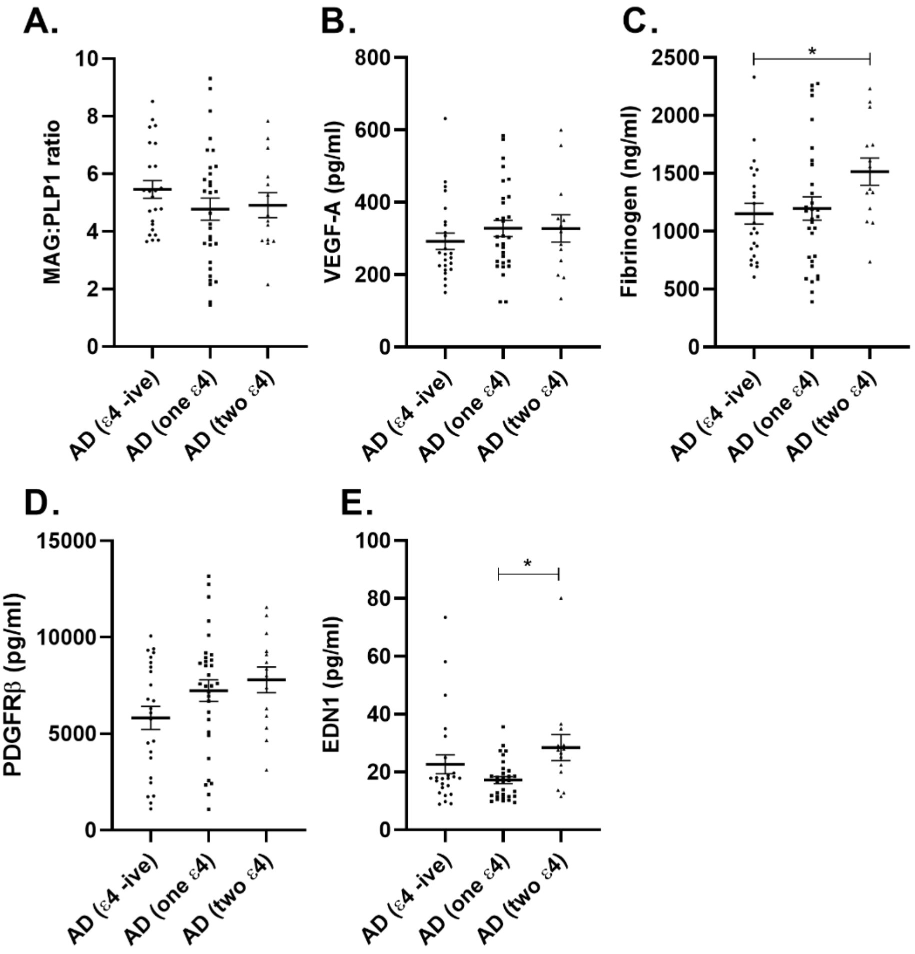

Supplementary Figure 8. Vascular markers in relation to *APOE* genotype. Scatterplots showing (A) MAG:PLP1 ration (B) VEGF (C) Fibrinogen (D) PDGFRβ and EDN1 (E) in AD cases (ε4 -ve indicates absence of ε4 and possession of either APOE ε2 or 3; one ε4 = heterozygosity; two ε4 = homozygosity). Each point represents the mean of a duplicate measurement for an individual. Horizontal bars indicate the cohort mean ± SEM. * $p < 0.05$

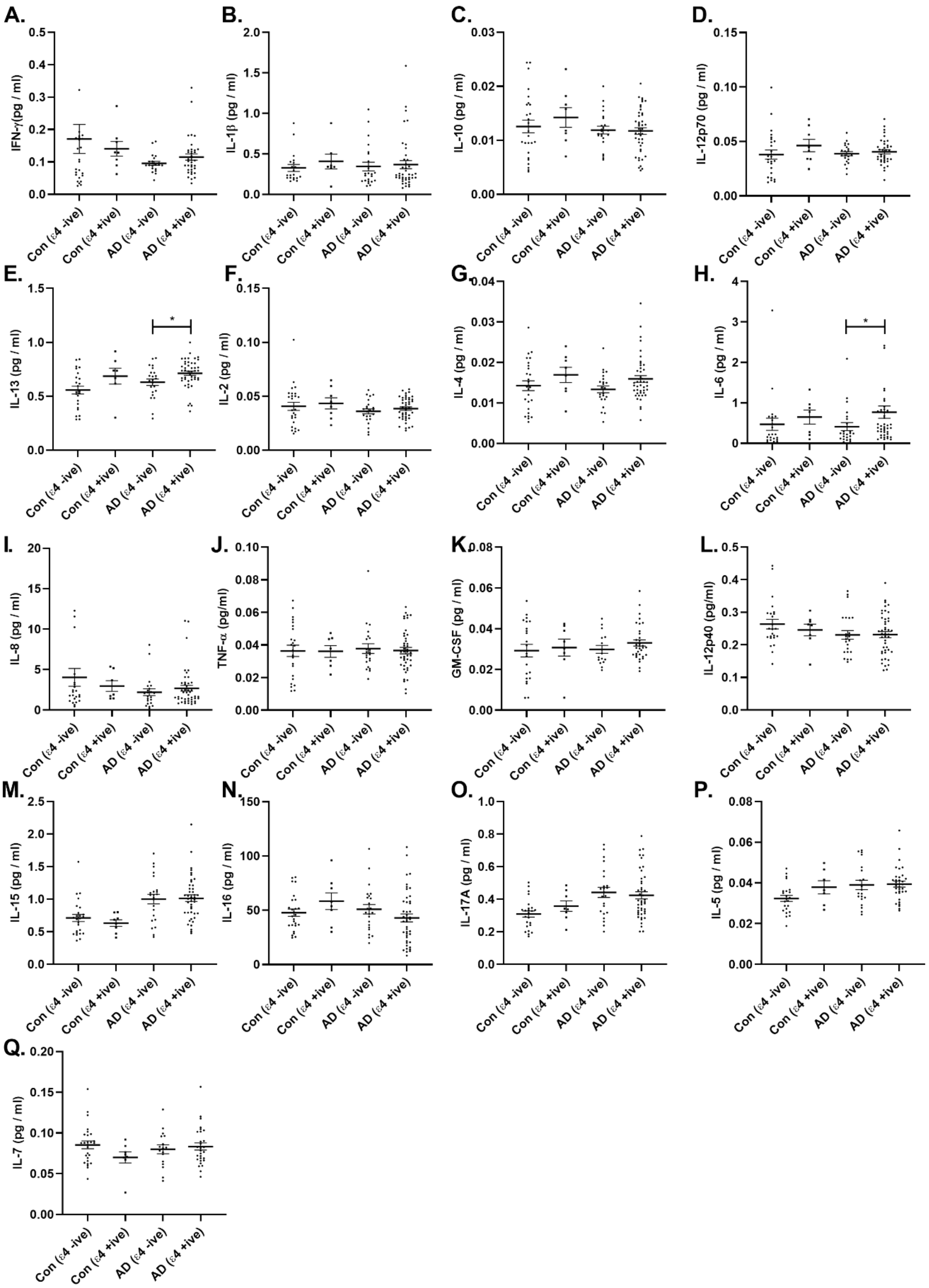

A.
IFN-γ(pg / ml)
B.
IL-1β (pg / ml)
C.
IL-10 (pg / ml)
D.
IL-12p70 (pg / ml)
E.
IL-13 (pg / ml)
*
F.
IL-2 (pg / ml)
G.
IL-4 (pg / ml)
H.
IL-6 (pg / ml)
*
I.
IL-8 (pg / ml)
J.
TNF-α (pg/ml)
K.
GM-CSF (pg / ml)
L.
IL-12p40 (pg/ml)
M.
IL-15 (pg / ml)
N.
IL-16 (pg / ml)
O.
IL-17A (pg / ml)
P.
IL-5 (pg / ml)
Q.
IL-7 (pg / ml)
Con (ε4 -ive)
Con (ε4 +ive)
AD (ε4 -ive)
AD (ε4 +ive)

Supplementary Figure 9. Brain cytokines in relation to *APOE* genotype. Scatterplots showing individual brain cytokine level (A-Q) in relation to presence (ε4 +ve) and absence (ε4 -ve) of *APOE* ε4 allele in the control and Alzheimer's disease cohort. Each point represents the mean of a duplicate measurement for an individual. Horizontal bars indicate the cohort mean ± SEM. * $p < 0.05$

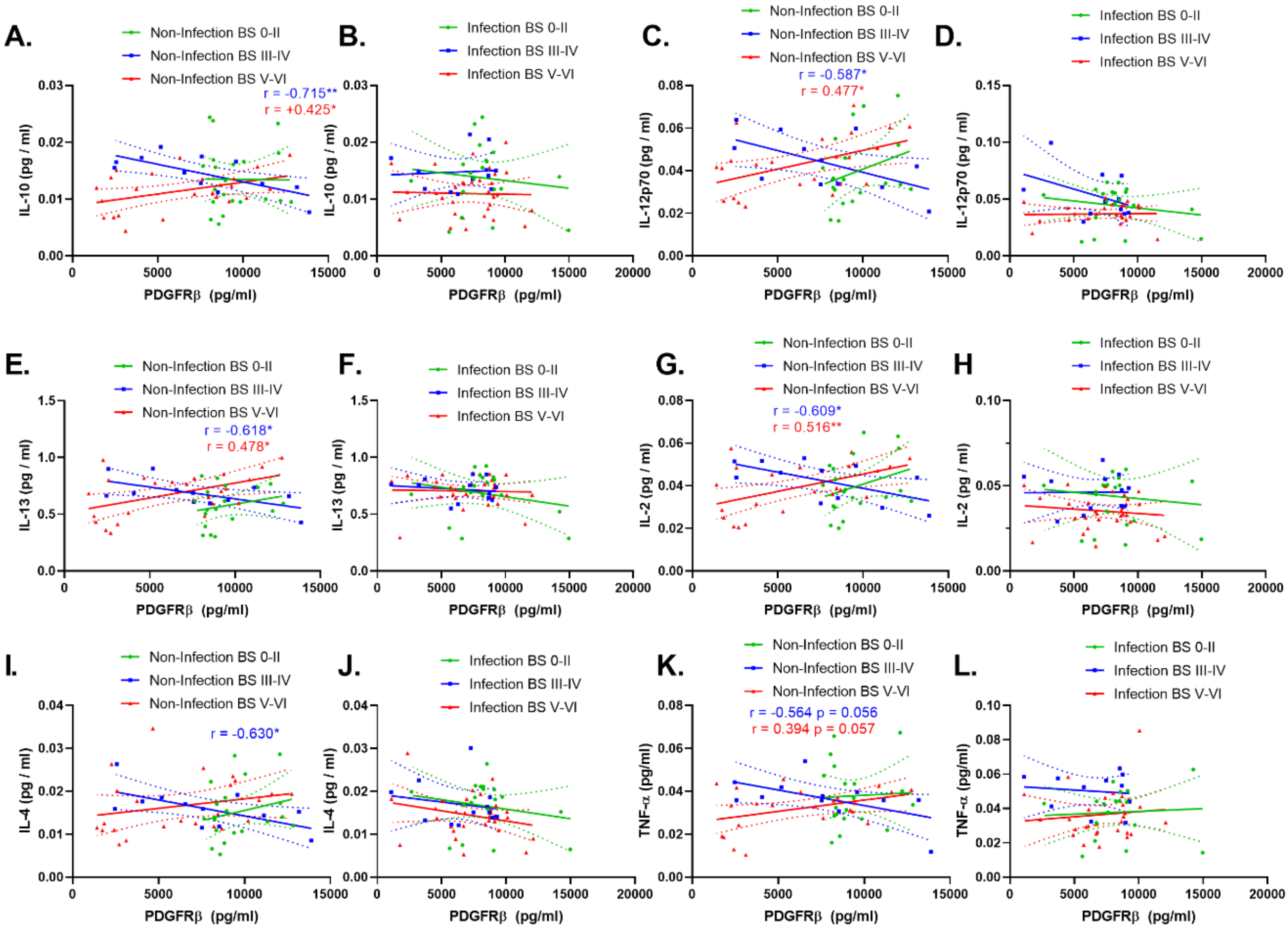

Supplementary Figure 10. Relationship between PDGFRβ and brain cytokines: influence of Braak stage and infection. In brains with intermediate tau tangle pathology (BS III-IV), PDGFRβ correlated negatively with several brain cytokines, but positively in BS V-VI, in cases without infection. This relationship as lost in cases with systemic infection. The best-fit linear regression lines and 95% confidence intervals are shown.

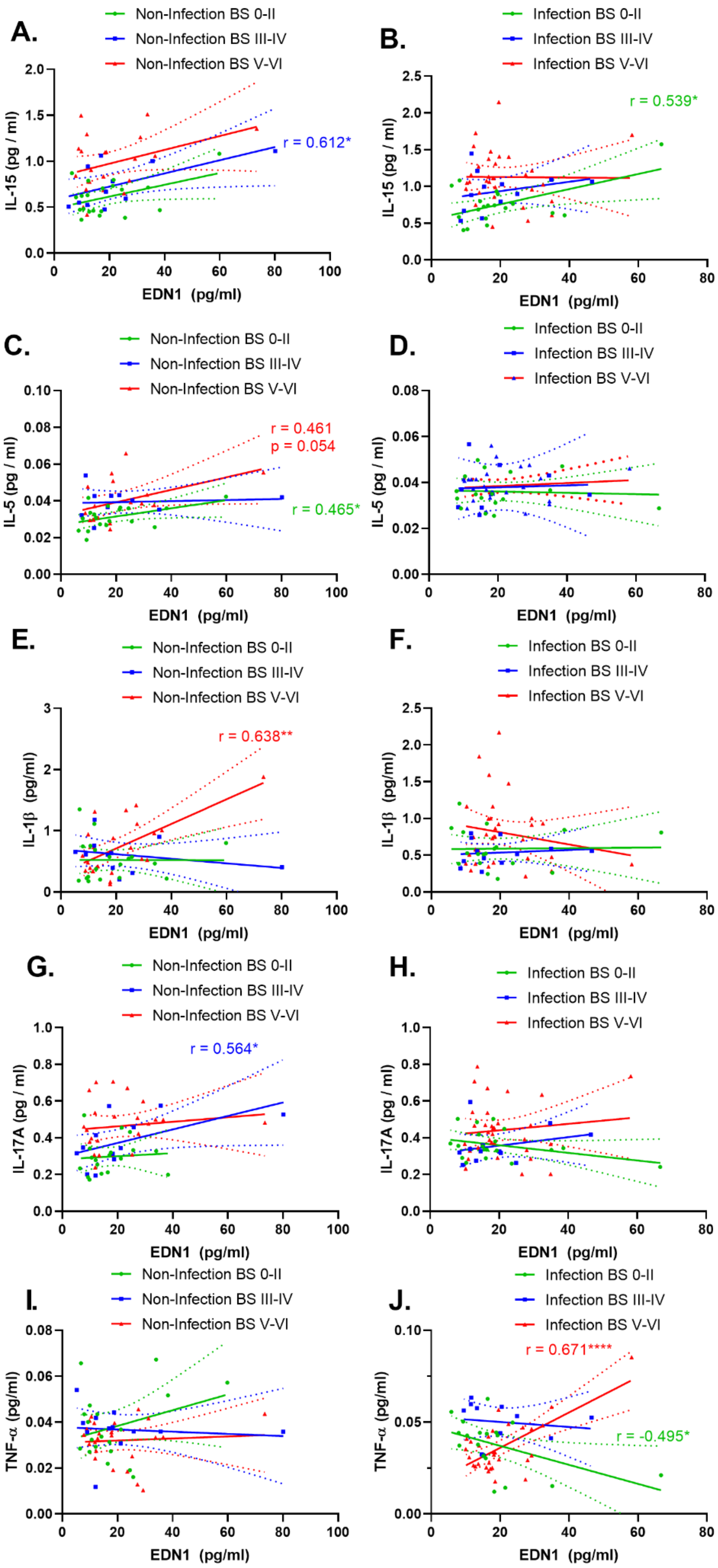
A.
Non-Infection BS 0-II
Non-Infection BS III-IV
Non-Infection BS V-VI
IL-15 (pg / ml)
r = 0.612*
EDN1 (pg/ml)
B.
Infection BS 0-II
Infection BS III-IV
Infection BS V-VI
IL-15 (pg / ml)
r = 0.539*
EDN1 (pg/ml)
C.
Non-Infection BS 0-II
Non-Infection BS III-IV
Non-Infection BS V-VI
IL-5 (pg / ml)
r = 0.461
p = 0.054
r = 0.465*
EDN1 (pg/ml)
D.
Infection BS 0-II
Infection BS III-IV
Infection BS V-VI
IL-5 (pg / ml)
EDN1 (pg/ml)
E.
Non-Infection BS 0-II
Non-Infection BS III-IV
Non-Infection BS V-VI
IL-1β (pg/ml)
r = 0.638**
EDN1 (pg/ml)
F.
Infection BS 0-II
Infection BS III-IV
Infection BS V-VI
IL-1β (pg/ml)
EDN1 (pg/ml)
G.
Non-Infection BS 0-II
Non-Infection BS III-IV
Non-Infection BS V-VI
IL-17A (pg / ml)
r = 0.564*
EDN1 (pg/ml)
H.
Infection BS 0-II
Infection BS III-IV
Infection BS V-VI
IL-17A (pg / ml)
EDN1 (pg/ml)
I.
Non-Infection BS 0-II
Non-Infection BS III-IV
Non-Infection BS V-VI
TNF-α (pg/ml)
EDN1 (pg/ml)
J.
Infection BS 0-II
Infection BS III-IV
Infection BS V-VI
TNF-α (pg/ml)
r = 0.671****
r = -0.495*
EDN1 (pg/ml)

Supplementary Figure 11. Relationships between endothelin-1 and brain cytokines: influence of Braak stage and infection. EDN1 correlated with several brain cytokines in cases with disease pathology (BS III-IV or BSV-VI) without systemic infection but not in cases with infection. The notable exception was TNF-α, which was strongly positively correlated with EDN1 in BS V-VI in the presence of infection. The best-fit linear regression lines and 95% confidence intervals are shown.

**Control (-)**	**Control (+)**	**AD (-)**	**AD (+)**	**VaD (-)**	**VaD (+)**
BBN_8671	BBN_8725	BBN_8848	BBN_8825	BBN_8861	BBN_8832
BBN_8684	BBN_8732	BBN_8852	BBN_8842	BBN_9313	BBN_4223
BBN_8691	BBN_8735	BBN_8915	BBN_8846	BBN_4208	BBN_9369
BBN_8706	BBN_8751	BBN_8968	BBN_8850	BBN_9387	BBN_14403
BBN_8708	BBN_8770	BBN_8990	BBN_8870	BBN_19611	BBN_19628
BBN_8709	BBN_8888	BBN_9118	BBN_8871	BBN_19622	BBN006.28583
BBN_8728	BBN_9028	BBN_9122	BBN_8892	BBN_24309	BBN006.29152
BBN_8739	BBN_9329	BBN_9189	BBN_8899	BBN_24310	
BBN_8835	BBN_9331	BBN_9198	BBN_8910	BBN_24901	
BBN_8898	BBN_9359	BBN_9200	BBN_8912	BBN_24904	
BBN_8923	BBN_4229	BBN_9274	BBN_8917	BBN006.26340	
BBN_8964	BBN_9392	BBN_9308	BBN_8978	BBN006.26572	
BBN_9292	BBN_9408	BBN_9309	BBN_9037	BBN006.31492	
BBN_9340	BBN_9422	BBN_9336	BBN_9076	BBN006.32513	
BBN_9344	BBN_19608	BBN_4200	BBN_9095	BBN006.33638	
BBN_4205	BBN_24337	BBN_4215	BBN_9109		
BBN_9354	BBN_24325	BBN_4216	BBN_9112		
BBN_9365	BBN_26009	BBN_9361	BBN_9123		
BBN_9407	BBN006.26096	BBN_4231	BBN_9150		
BBN_19613	BBN006.28893	BBN_4232	BBN_9156		
BBN_22623	BBN006.29470	BBN_9367	BBN_9162		
BBN006.29018	BBN006.31516	BBN_9371	BBN_9188		
BBN006.30165		BBN_9375	BBN_9242		
BBN006.30198		BBN_9377	BBN_9243		
		BBN_9378	BBN_9263		
		BBN_9395	BBN_9266		
		BBN_9417	BBN_9269		

		BBN_9420	BBN_9295		
		BBN_9426	BBN_9296		
		BBN_10252	BBN_9315		
		BBN_19615	BBN_9317		
		BBN_24330	BBN_9323		
		BBN006.28710	BBN_9326		
			BBN_9338		
			BBN_9341		
			BBN_9343		
			BBN_9346		
			BBN_9372		
			BBN_9379		
			BBN_9401		
			BBN_9433		
			BBN_19614		

Supplementary Table 3. List of MRC UK-BBN identifier numbers for cases used in this study. Controls (Con-/Con+), Alzheimer's disease (AD+/AD-) and Vascular dementia (VaD+/VaD-) with (+) and without systemic infection (-)
